# A solid waste classification using reinforcement learning boosted by data augmentation and hyperparameter optimization

**DOI:** 10.1038/s41598-026-46347-7

**Published:** 2026-04-19

**Authors:** Kavyan Maleknya, Roohallah Alizadehsani, Siamak Pedrammehr, Kimia Shirini

**Affiliations:** 1 Department of Civil and Environmental Engineering at Politecnico di Milano, Milan, Italy; 2 Institute for Intelligent Systems Research and Innovation, Deakin University, Geelong, VIC Australia; 3 Faculty of Design, Tabriz Islamic Art University, Tabriz, Iran; 4 Faculty of Multimedia, Tabriz Islamic Art University, Tabriz, Iran

**Keywords:** Solid waste management, Active learning, Deep reinforcement learning, Data augmentation, Hyperparameter optimization, Engineering, Environmental sciences, Mathematics and computing

## Abstract

With the rising rate of urbanization and the high rate of development of smart cities, handling the increasing amount of waste, both in terms of their production, sorting, and discarding, has become an urgent task. New plans have used the convolutional neural network (CNN) techniques, and huge pre-labeled image data to enhance the control of garbage including sorting, dump, disposal and recycling. Nonetheless, formation and labeling of such datasets is also time and cost demanding. In order to solve this problem, the proposed study aims to use an active learning (AL) framework that would enhance categorization of waste types, including organic, and recyclable, with less labelled examples. Normal models of AL tend to apply fixed selection mechanisms, which are not able to be adjusted to evolving data. The model combine the deep reinforcement learning (DRL) with an innovative scope loss function (SLF) to improve adaptability. The purpose of this step is to balance the trade-off between exploration and exploitation of new information. Besides, online data augmentation is performed with the help of a generative adversarial network (GAN). An additional regularization approach is incorporated in order to make GANs training more stable and reduce the likelihood of mode collapse. Moreover, a better algorithm of hyperparameters tuning (pattern) is applied and a k-means mutation technique is employed to select the most suitable ones. The efficiency of the proposed method is proved on three benchmark datasets, namely TrashNet, Trash, and OrgalidWaste. The active learning process starts with only 10% labeled samples, while the remaining 90% are treated as unlabeled. At each iteration, the DRL agent selects a small batch of informative samples under a fixed annotation budget. The classifier is retrained incrementally after each selection round until the labeling budget is exhausted. F-metrics of the model are 92.29, 89.27 and 89.90, indicating distinct advancements in waste classification using images and few labelled data. Generally, this research would help in creating a more intelligent and efficient wastes management systems.

## Introduction

 The rapid technology expansion in urban areas has resulted in various issues among individuals and one of the greatest problems is how to handle the solid waste^1^. Individual consumption of waste produced in cities daily has been averaged at 0.74 kg on individual city basis. By the year 2025, the amount of waste generated throughout the world is going to hit approximately 2.2 billion tonnes^2^. There are numerous types of waste produced by this, which include, but are not limited to, rubber, metal, food scraps, fabrics, glass, plastics, paper, and electronics, healthcare, and cleaning product types. These can be separated into two major categories, which include organic and recyclable. It has been demonstrated that nearly all garbage collected is made out of daily items such as leftover foodstuffs, paper and glass that is almost 99.5% of the total garbage. This demonstrates the presence of both dry and wet wastes in big proportions. The categorization of waste by whether it is biodegradable or not is a way to enhance recycling schemes and disposal. It is due to this that a number of researchers have begun to concentrate more on this domain in the recent years^3^.

Over the last few years, CNNs and their variants have gained much relevance in enhancing the solid waste identification accuracy^1,4^. Such networks are capable of learning in an efficient way layered and unique visual features of images. They, however, depend on large datasets with accurate annotations, which are both expensive and time-consuming to mix^5^. Due to this reason, there is a need to reduce the cost of labeling of image or come up with a mechanism that will be able to train models with only a small number of annotated samples^6,7^. AL copes with it by naming only the informative unlabeled data. This model is consequently trained on the labeled data samples. Nevertheless, traditional AL methods usually rely on a set of hard-wired rules, such as representativeness or measures of uncertainty^8^. These measures fail to change with the changes in understanding of the model when it is trained. As one example, a data point that is initially complex to determine can be made very easily determined later. Unchanging approaches cannot detect this shift, hence, ineffective data selection, unwarranted labeling, and slower learning process. These constraints are notably apparent in CNN-based waste classification, during which the dynamics of the model vary between the various stages of learning^9^.resolves these challenges by modifying its choice of data to select during learning. DRL is not based on fixed rules. It emulates the responses of the model to decide the usefulness of labeling each sample and continues to change its approach^10^. Unclear samples that could later be predicted may be left. This reduces repetition of labels and the system is able to focus on more valuable data. It is a flexibility that enhances the labeling quality, and also quicker model training. There are, however, some drawbacks to DRL-based systems: locating a balance between exploitation and exploration, retaining the capacity to make generalizations, and effective tuning of complex hyperparameters^11^.

Locating the appropriate trade-off between exploitation and exploration in DRL is a major determinant of the stability of the performance When a model is over-targeted that may be overly restrictive, it experiences weak or incompetent strategies. Some new practices have attempted to control this issue. For example, adaptive entropy regularization regulates the exploration rate by adding entropy rewards whenever needed. The approach of goal-achievement directed exploration (GAGE) upsets such balance depending on the achievement attained by the agent in meeting its goals^12^. The other method is referred to as SI2E, and it is an exploration process that makes use of structural mutual information to ascertain the pattern within state-action spaces without interfering with important spatial structures^13^. Despite the fact that these techniques enhance the efficiency of learning as well as policy robustness, each shows some shortfalls. Adaptive entropy frequently requires and responds responsibly to hyperparameter adjustment, and can do poorly on variable environments. GAGE is also flexible but fails to evaluate task achievement in the low-density choosing of rewards. SI2E, however, makes the computations process more expensive and incompatible with active learning schemes. This paper presents an SLF, which provides more efficient exploitation and exploration alone. SLF does indirectly encourage exploration through minimizing the propensity of a decision-maker to over-rely on high confidence judgments. It also restricts the impact of gradient changes which overweight some predictions. SLF ensures a variation in decision-making and reduces the odds of the practitioners reaching suboptimal policies early by scaling gradient magnitudes based on action confidence.

The idea of data augmentation assists DRL in diversifying the data, balancing classes, and accomplishing a better generalization of the model in adaptable study environments^14^. It has been divided into online and offline approaches. The offline methods add additional data prior to the commencement of training that augment the amount of computation, as well as the extension of the training time. Online techniques, in contrast, create new samples on the fly and ensure that the training machine maintains the dataset size constant and decreases storage needs. Online augmentation has been extensively supported with the use of GANs that would help to enhance generalization^15^. Nevertheless, they also usually have certain issues, such as unstable training and mode collapse, that decrease the diversity of produced samples. In order to overcome these shortcomings, a number of approaches have been devised. Auto-encoding GANs^16,17^ employ series of generators and encoders to increase the variety of feature and characterization, especially in problems such as medical imaging. Alternative methods have also been used that are aimed at stabilizing gradient flow and minimizing mode collapse, such as identity blocks^18,19^ in models like deep convolutional GAN (DCGAN) and auxiliary classifier GAN (ACGAN)^20^, Though such methods may have a positive effect, they also have the tendency to complicate the networks, increase their training processes, and restrict their ability to embrace various datasets. A GAN is suggested in this study, which is combined with a selective gradient omission mechanism to address instability and mode collapse. This design does not introduce structural complexity, instead, it narrows down the processing of learning by eliminating less meaningful gradients and training only meaningful updates. This makes the outcome a more stable model that will produce a broader range of high-quality samples to augment on-line resulting in superior generalization and higher truth to the solid waste classification task.

Hyperparameter optimization is a vital part of DRL as it depends on learning regularity, convergence velocity, and model precision^21^. A range of methods have been suggested to control the hyperparameters sensitivity of DRL, such as grid search^22^ and genetic algorithms^23^.The approach of grid search considers all combinations of parameters to find the most effective ones, however, it is very resource and time consuming. The genetic algorithm is based on the imitation of the natural evolution and involves the process of enhancing the candidate solutions by selection and variation. However, they are vulnerable to fluctuating convergence, and are not always able to achieve optimum results. The solution to this problem is found in the more flexible metaheuristic optimization methods^24^. One of them, the DE algorithm, has a fair trade-off between general search and local optimization. Its performance however declines as mutation and crossover cannot be adequate to search new regions or when the algorithm is overly converged due to strong selection pressure. In order to improve DE, this paper uses human mental search (HMS) reasoning and k-means-based clustering as part of the optimization process. This enhanced edition enhances efficient correspondence among prospective solutions, global searching, and local refining steadiness. It also reduces the chances of falling into local minima and is more certain to deliver better optimization results. Recent feature extraction strategies in intelligent systems—including ensemble learning^25^, metaheuristic-optimized recurrent networks^26^, and attention-enhanced architectures^27^—have demonstrated significant performance gains in specialized tasks. However, limited attention has been devoted to understanding how feature distributions evolve under reinforcement-driven active learning frameworks, particularly in the context of waste classification where labeled data is scarce.

This study presents an AL framework to solid waste classification based on DRL with an SLF and a gradient-omission-based GAN of online data augmentation and hyperparameter optimization with HMC. Its aim is to transform three key challenges, namely adaptive sample selection, better generalization and useful hyperparameter optimization. The proposed system uses multiple CNNs in order to obtain multi-level image features. The processing of these elements is followed by a softmax layer to do the classification. The first phase of the training is based on a small amount of labeled samples. With DRL, unlabeled data is being constantly checked during the training process and the samples that should be included into the labeled data are identified. The selection round is repeated with the retrained classifier with the new set of data each time. This process proceeds in an iterative manner and creates a continuous learning process, in which new samples are selected and labeled according to preset selection policies. The strategy approximates automatic sequential decision-making and thus enables the model to continually update its knowledge and improve classification accuracy with only a small amount of labeling.

The general contributions of the model can be discussed as follows:

- The model also finds the assistance of DRL when choosing unlabeled data that will benefit the learning process the most, using a more advanced form of AL.
- In data augmentation, the model uses a specialized GAN that is used to perform augmentation on-line. This capability allows the model to complement diverse scenarios because it produces realistic synthetic images to train, so there is no necessity of obtaining extensive datasets in the real world to do so. Another new regularization technique is also utilized to make GAN training stable and prevent mode collapse to safeguard the formation of different and dependable training cases.
- The model applies a better DE approach to optimize hyperparameters with the mutation phase advised by k-means clustering. This can help the DE algorithm to effectively search in the hyperparameter space and find the best settings by stressing on favorable clusters of solutions. This method improves the tuning process by making it specific to the requirements of the model, whereby all the parameters are adjusted so as to provide high accuracy and performance.

The structure of the given paper is as follows: Section  “Related works” provides a detailed analysis of the relevant research, which preconditions the introduction of the methods. In Section  “Materials and methods”, the proposed model strategy and design are looked at. Section  “Empirical evaluation” provides an account of the findings of our empirical evaluations and their consequences. Section “Conclusion” at the end of the paper is a conclusion that summarized the main learnings and discussed the perspectives of further research.

## Related works

In recent years, various techniques have been developed to classify solid waste, encompassing machine learning, deep learning, and models based on transfer learning. Each of these categories is thoroughly examined in the subsequent sections of this paper.

### Machine learning

Mezza et al.^28^ considered how the field of artificial intelligence (AI) can be used to facilitate improved decisions when managing municipal solid waste (MSW) in 2023. They contrasted two approaches, which are support vectors machine (SVM) and the long short-term memory (LSTM) networks. The SVM was also tested in the paper to determine whether it suits particular kind of data. It also applied LSTM together with varying configurations and time-based filters to examine annual trends in waste collection. Tao et al.^29^ became preoccupied with challenges of locating thermochemical properties and eliminating inorganic substances in MSW using common methods. Their study proposed an algorithm that can connect hyperspectral imaging to machine learning. This was aimed at saving on time, steps and samples. Its goals were to enhance the quality of inorganic portions detection and to estimate hydrogen, carbon, nitrogen, oxygen, as well as the low heating value (LHV) of organic waste. Important parts of spectral data were extracted using principal component analysis (PCA), whereas the classification and prediction were done with the aid of artificial neural networks. Adeleke, et al.^30^ developed an adaptive neuro-fuzzy inference system (ANFIS) that has been optimized using two evolutionary calculators genetic algorithm (GA) and particle swarm optimization (PSO). Their model dealt with the issue of poor data collection when it comes to waste studies. The research involved seasonal variations of solid waste in Johannesburg employing three clustering methods, namely subtractive clustering (SC), grid partitioning (GP) and fuzzy c-means (FCM) with different combinations of parameters. This study demonstrates how AI can be used in modeling physical properties of waste, which is useful in advancing the waste management plans. Shah et al.^31^ have also contributed to resolving the mounting challenges in solid waste management in urban India by adopting an approach to categorize seven classes of solid waste into biodegradable and non-biodegradable. The method used is on both training and testing dataset images, it uses features in a form of hybrid features (HF) to extract features of the image; it uses MeQryEP and principle component analysis ( PC ) to extract texture and shape. The identified features are combined and processed by a deep neural network (DNN) to classify the waste, which is much more precise than the traditional methods such as PCA with different algorithms.

Abu-Qdais et al.^32^ attempted to promote sustainable waste management by analyzing solid waste using automated solid waste classification in 2024. The current paper presents an assessment of the effectiveness of conventional and deep machine learning algorithms in waste sorting. The traditional techniques like random forest (RF) and SVM have been compared to a deep learning CNN. Moreover, to recognize six types of solid waste, a new JONET deep learning model, based on a pre-trained DenseNet 201 and a new architecture with a 1024-neuron fully connected layer, is constructed. This approach uses publicly available and custom-created data to train and test the model, giving more attention to automated strategies to maximize classification accuracy and minimize the risk of worker safety. Fatovatikhah et al.^33^ resolved the urgent requirement of fast and precise prediction of the flood behaviour with the rising extreme precipitations as a result of the climate change. This paper takes an LSTM and SVM method in order to effectively forecast flood waste. Two separate raw data based on the article Advancing Sustainable Materials Management: Facts and Figures 2015 that covers nine years (1960–2015) are displayed on the generated and recycled materials of the municipal waste stream. The article groups wastes as different types of waste; they include rubber, food, metals, textiles, yard trimmings, wood, plastics, glass, paper, or any other wastes that fall under miscellaneous inorganic waste to enhance flood management and recovery.

Principal issues surrounding the application of machine learning solutions in solid waste classification are due to several drawbacks of the traditional machine learning algorithms. To begin with, the machine learning models tend to require complex selection of features and expert knowledge to locate significant patterns in complex data. Such preprocessing may consume some time and may also fail to recognize the fine differences in various waste substances hence it produces less precise classifications. Secondly, conventional machine learning algorithms frequently require scalability and efficiency assistance, especially when the data at hand comprises massive solid waste, and this amount increases indefinitely with the increasing quantity of waste data. The methods typically cannot handle large sets of features and excess variables can reduce accuracy and efficiency of the model. Moreover, it is commonly often convoluted and multitrial to fine-tune the parameters and settings of many machine learning algorithms, which may contain many trials and errors.

Deep learning has been used to solve a number of problems because it takes advantage of automatically learning features, which eliminates the requirement of manual work. They can be applied to big data and complicated feature space. The CNNs are also applicable to spotting spatial patterns of an image, a process that can identify different types of waste which are metal waste, plastic waste and garbage.

### Deep learning

The application of deep learning to sort solid waste has been mentioned in a number of studies, and it has indicated the growing importance of the method and its prospects in the field of waste management studies. Li et al.^2^ suggested the use of CNN, Graph-LSTM, two types of deep-learning methods, to solve the problem of recycling and waste management in developing nations where municipal solid waste is increasingly problematic. The article uses a convolutional neural network to detect six types of waste objects (plastic, cardboard, organic, metal, paper, and glass) on the belt conveyors of waste collection systems. Thanks to the visual combination of Graph-LSTM, long-term dependencies within the system are improved, which promotes its performance and generalization, and as a result, more efficient and independent waste management. Niu et al.^34^ resolved the problems of the solid waste site detection in urban areas by the operation of a new deep learning model that integrates a multi-scale dilated CNN and a swin-transformer. This model succeeds in the comprehensive (local) and coarse (global) details, where high-resolution remote sensing images depict solid waste, allowing the mapping of solid waste better in different cities with no reference pixel-based labelled data. A model strategy indicates that there has been an important evolution in the management of waste in the cities in the way of effective aggregation of different spatial features. A model by Prakash et al.^35^ was presented to predict municipal waste with a tree-based deep CNN that was enhanced with a hybrid optimization procedure to increase accuracy and reduce the amount of time spent on processing this feature. The results were obtained through morphological filtering and empirical wavelet procedures in cleaning up of the data prior to the application of the proposed algorithm, which was conducted in various regions of Chennai. The model was written in Python and was created to be able to predict several types of waste that would occur between the years 2025 and 2035. Its findings were superior and quicker computation than at the previous methods. Yudhana et al.^36^ analyzed the application of machine learning in waste problem solution, especially sorting waste materials in the world. They constructed a CNN model in order to classify waste in a better way, involving better data preparation and a relatively simple structure to enhance results. The model was experimented on a Kaggle dataset and demonstrated a high promising potential in ranking various types of waste. This will reduce landfill, will preserve nature and conserve resources through a better accuracy of separating waste.

Jayaraman et al.^37^ described a layered model to detect municipal waste with automation in 2024. It performs its own custom CNN and a MobileNet that is optimized with Bayesian optimization with gradient boosting serving as the final classifier. Such an approach will aid in enhancing waste sorting, reduce human participation, reduce health risks, and allow city services in better ways of managing waste. It was proposed by Murugan et al.^38^ that a new approach should be used that employs CNNs combined with an easy-to-use HTML and Django framework to advance solid waste management (SWM) practices. This method will also improve waste prediction and classification as a user can conveniently sort and designate various types of waste (such as non-biodegradable and biodegradable) to a single type. The system employs cutting-edge deep learning technology and straightforward interfaces to suggest enhanced waste disposal, benefiting the promotion of eco-friendly solutions and the advancement of environmental protection solutions. The article by Chen et al.^39^ investigated how enhanced data classification through noise can improve the accuracy of municipal solid waste (MSW) classification with the aid of computer vision. The present research compares two state-of-the-art classification algorithms, CNN and deep residual shrinkage networks (DRSN), in terms of their ability to solve the problem of small quantities of samples and feature imbalances in MSW images. Through the noise-assisted data enhancement techniques, the study will enhance the model training and also increase the model robustness to noisy data. This strategy is very vital in improving the effectiveness of MSW classification systems when used in industries.

Numerous deep learning-based methodologies have been published in literature, most of them utilize CNNs to classify solid waste materials. Such approaches, though, can be enhanced further because the vast majority of them demand a substantial amount of pre-labeled image collections, which are rather expensive and time-intensive. Other issues involve the fact that methods that are based on deep learning are limited to generalizability. Due to this most of the models develop well in the datasets that it was trained in and is normally poor in case it is introduced in different datasets. This poses a serious constraint since such models are supposed to be applicable in different situations that are dynamically varying in such an area like solid waste. Hyperparameter adjustment issues that are based on manual tweaking are other common challenges in l deep learning strategies, as they are not optimal. Hyperparameter tuning is also crucial since it has a significant impact on both the model training and its performance.

To address these issues, we will implement a combination of DRL and an SLF to select strategic data, a better GAN to increase the generalizability, and an improved DE algorithm to optimally set the hyperparameters. We would aim to advance the toolkit on solid waste classification based on a theoretical, innovative approach, that is also practically flexible to implement in a number of operational situations in the real world.

### Transfer learning

Due to the paucity of the available data, studies have come up with ways of increasing the number of datasets. Zhang et al.^1^ have suggested in 2023 a way to segregate the waste in any urban city to identify areas to be recycled and those to be organic, in order to enhance smart city waste systems. They use data preparation, AlexNet based feature extraction, classification using a deep belief network (DBN) and fine-tuning using Optuna. The model improves the level of prediction and enables one to design better waste management strategies. The different pre-trained CNN methods were compared by ALALIBO and NWAZOR^40^ to classify solid waste that could be recycled on the basis of MATLAB platform. Eight CNN architectures are evaluated in the study; they include ResNet50, AlexNet, EfficientNetB0, ResNet18, Inception V3, MobileNet V2, GoogleNet, ResNet101. All models were trained with 5, 7, and 10 epochs on the TrashNet dataset, which was divided into a training and a validation section (75 and 25% respectively). Such strategy highlights the fact that CNNs are effective in improving accuracy of the waste classification systems, which is paramount to sustainable processes of waste management and recycling. Srilatha et al.^41^ proposed a two-step CNNs solid waste detection and classification approach. Their treatment assists in the enhancement of intelligent waste management. The model is developed on ResNet101 that extracts important features based on a set of convolution layers of input images. Over such layers, a region proposal network (RPN) is used. It generates 256-value feature vectors, estimates the probability of the presence of objects, and creates the approximate initial bounding boxes of the potential waste objects. These options are finally sent to a softmax regressor and a box regressor. They combine in assisting the model to identify and find five types of waste, namely glass, plastic, cardboard, paper, and metal. It is based on the more rapid RCNN design. It also does not employ any selective search as older R-CNNs, but rather the RPN to scan the feature maps. The processing time is lowered and object detection accuracy is increased and hence, the process becomes more efficient. Kaya et al.^42^ introduced a model in automatic waste classification optimization of hyperparameters like depth and width for adaptation of waste management systems in cities. The authors applied a 6-layer CNN and VGG19, DenseNet169, and other models, which are current, with transfer learning and fine-tuning methods. Many experiments using the GridSearch contributed to finding the best settings and improving the accuracy and F1 scores significantly and moved the waste classification to the automation stage. Ramya et al.^43^ paid attention to the acute issue of waste management in growing smart cities in India. They suggested a novel approach with the application of convolutional neural networks. The model utilizes the ResNet-152 and the MobileNet V2 models. The networks are both enhanced with the help of transfer learning to increase their effectiveness in waste classification problems. These models are intended to examine, categorize, and enhance the separation procedure of solid waste, which impacts on the eco-sustainability of open cities. The application of CNNs in the waste management is a promising demonstration of the opportunities to cause the decrease in the level of environmental impact and the development of smart cities. Jose et al.^44^ discussed the increasing challenges about the management of waste in cities due to the increasing urbanization and population. Their model presented a two-branch Faster R-CNN model/complex-range seeking algorithm optimization system to sensible sorting of wastes. They had experimented this framework in some of the largest cities in Maharashtra. Accuracy, recall, and precision, and F-measure were the assessment criteria. The results indicated that the offered system is superior to the previous classification methods. Alrayes et al.^45^ came up with a novel approach of sorting waste automatically with the help of a vision transformer and a multilayer hybrid CNN. The issues that were witnessed in the previous systems such as slow speed and inaccuracy are duly addressed in the proposed model by designing the network more thoroughly and improving the links between modules. This begins by collecting image data, deriving essential characteristics, and a process of normalization. It is aimed at adjusting the classification settings of waste images and selecting the most effective one. This enhanced system generates a less complicated yet more precise model of determining the different types of waste. Jin et al.^46^ came up with a better MobileNetV2 model which incorporates an attention mechanism to enhance the accuracy and speed of waste detection. This is relevant in successful recycling. Transfer learning has an advantage on the model in that pre-trained weights can be used to enhance generalization. PCA is also used to improve processing speed on edge equipment. A prototype constructed using this technique demonstrates significant improvement in accuracy and performance, providing a good alternative of an automatic sorting waste system. Yong et al.^47^ addressed the issue of the low household waste separation with the help of a MobileNetV2 deep learning model. In their approach, household garbage is subdivided into 4 categories including recyclable, hazardous, kitchen, and general waste. MobileNetV2 is faster and more accurate when compared to previous CNN models. This leads to a better classification of the waste and a reduction in time and effort that have to be captured manually. The model can also be used on mobile devices as it is lightweight and this makes it a useful tool of automatic sorting of wastes.

In 2024, Lilhore et al.^48^ explored ways to improve waste handling processes by designing a Hybrid CNN-LSTM model that supports smart city goals. Their approach combines convolutional and recurrent neural networks, using transfer learning to improve how waste is sorted, composted, collected, and disposed of. The model focuses on classifying waste into two groups: recyclable and organic. To overcome problems like overfitting and small dataset size, they applied transfer learning using ImageNet and added data augmentation strategies. The performance of the model was evaluated using the TrashNet dataset, which contains over 27,000 labeled images. Several CNN models, including ResNet-34, VGG-16, AlexNet, and ResNet-50, were tested. These architectures were trained over several epochs and optimized with the adaptive moment estimation (AME) algorithm. Performance was measured using accuracy, recall, precision, and both training and testing loss to ensure a fair comparison and identify the most effective configuration. Santoso et al.^49^ developed a new system for classifying waste by combining a pre-trained EfficientNet model with PCA to reduce feature dimensions. The method uses transfer learning for strong feature extraction through the EfficientNet-based CNN. PCA is then applied to refine and reduce the size of the extracted features. This system merges outputs from convolutional and average pooling layers before sending them into a fully connected layer for final classification. The model was tested on a labeled waste dataset and showed much better results than previous techniques. Its success highlights the benefits of combining CNN with PCA for improved accuracy and efficiency. Future improvements could include testing on larger datasets and applying the model in real-time scenarios.

While transfer learning is generally more efficient with data than standard deep learning methods, it still faces several limitations that can be mitigated through AL techniques. One major difficulty is domain adaptation. A model trained on one dataset often performs poorly when tested on a different dataset with distinct properties. AL addresses this problem by carefully selecting and labeling the most informative samples from the target domain, allowing the model to adjust more effectively to new patterns. Another issue is catastrophic forgetting, in which the model slowly forgets what it learned from the source data while learning from new inputs. AL helps control this effect by maintaining a balance between old and new samples, ensuring that important knowledge from the source remains while the model adapts to new information. Another difficulty is prediction bias, which happens when the source and target data are not well matched. AL helps enhance both fairness and stability by regularly selecting varied and representative samples from the target set. This process strengthens generalization across varied conditions. Through these mechanisms, AL supports the efficient training and dynamic fine-tuning of transfer learning models, enabling better performance in evolving data environments.

### Advanced feature extraction strategies in intelligent systems

Recent advances in intelligent systems have demonstrated that performance gains are often strongly influenced by effective feature extraction strategies rather than classifier design alone. For instance, in the context of smart energy systems, Hussain et al. in their study titled Using machine learning ensemble method for detection of energy theft in smart meters employed ensemble-based learning to enhance discriminative feature diversity in imbalanced environments^25^. Their results indicated that feature-level diversity significantly improves robustness against noisy measurements. Similarly, Simfukwe et al.^26^ proposed a metaheuristic-optimized architecture in Enhanced human motion detection with hybrid RDA-WOA-based RNN and multiple hypothesis tracking for occlusion handling to refine temporal feature representations under complex occlusion conditions. In the domain of medical image analysis, Wang et al.^27^ introduced specialized attention mechanisms in Enhanced U-Net with Attention Mechanisms for Improved Feature Representation in Lung Nodule Segmentation, demonstrating that spatial attention modules substantially improve feature saliency and segmentation performance. These studies collectively highlight the importance of adaptive feature extraction mechanisms within supervised learning paradigms. Unlike the aforementioned approaches, which enhance representation primarily through architectural modifications, the proposed framework in this study dynamically reshapes the feature distribution by controlling sample acquisition via DRL-guided active learning. Table 1 shows the Comparison of recent feature extraction strategies across different domains.

**Table 1:** Comparison of recent feature extraction strategies across different domains.

Study	Application domain	Feature extraction strategy	Learning paradigm
Hussain et al.^25^	Smart Grid	Ensemble-based feature diversity	Supervised
Simfukwe et al.^26^	Motion Detection	Metaheuristic-optimized temporal features	Supervised
Wang et al.^27^	Medical Imaging	Attention-enhanced spatial features	Supervised
Proposed Framework	Waste Classification	DRL-guided dynamic feature evolution	Active Learning

## Materials and methods

Lilhore et al.^45^ also conducted research on the optimization of waste processing by developing a Hybrid CNN-LSTM model that facilitates the objectives of smart cities in 2024. They use a mix of the convolutional and recurrent neural networks and transfer learners to enhance the process of waste sorting, compost, collection, and disposal process. The model is based on the grouping of waste into two categories, namely recyclable and organic waste. In order to address such issues as overfitting and insufficient size of the dataset, they used the transfer learning on ImageNet, and implemented data augmentation options. TrashNet dataset which comprises more than 27,000 labeled images was used to test and evaluate the performance of the model. A number of CNN models such as VGG-16, ResNet-34, AlexNet, and ResNet-50 were experimented. Such architectures have been trained across multiple epochs and with the adaptive moment estimation (AME) algorithm. Accuracy, recall, precision, training and testing loss were used to measure performance to guarantee that a fair comparison was made and the best setup was determined. The Santoso et al.^46^ enhanced an entirely new system of categorizing waste using a pre-trained EfficientNet model and PCA to downsize the features. The approach applies a transfer learning approach to extract powerful features by using EfficientNet-based CNN. PCA will then be used to narrow down refine and scale down the extracted features. This framework combines convolutional and average pooling layer output after which they are channeled into a fully connected layer to be finally classified. The standard was tried on a tagged waste dataset and classified much higher than a prior technique. The success of it indicates the excellence of a CNN in conjunction with PCA to achieve better accuracy and efficiency. To be improved in the future, additional testing with bigger data sizes and implementing the model to real-life situations should be conducted. The DRL component is implemented using a Deep Q-Network (DQN), a value-based reinforcement learning algorithm suitable for discrete action spaces. We employ a Deep Q-Network (DQN) as the reinforcement learning backbone. PPO is not used in the final implementation.

Although transfer learning tends to be more effective with data as compared to conventional deep learning approaches, it has a number of limitations, which can be resolved with the help of AL methods. Domain adaptation is one of the major challenges. The performance of the model that is trained on one dataset is usually low when the model is tested on a new dataset that possesses unique characteristics. AL solves this issue by being selective and labelling the most informative samples in the target domain to enable the model to adapt better in new patterns. The other problem is catastrophic forgetting whereby model gradually forgets what it was taught by the source data as it continues to learn new inputs. AL assists in regulating this influence by keeping the balance between the old and the new samples so that the valuable information of the source is preserved and the model gets updated based on the emerging information. The other challenge is prediction bias that occurs when the two data, source and target are mismatched. AL assists in promoting fairness and stability through frequent selection of diverse and representative samples among the target set. This enables generalization in diverse circumstances. AL facilitates effective training and active fine-tuning of transfer learning models at the expense of these mechanisms, which allow improved performance in changing data settings.

**Fig. 1:**
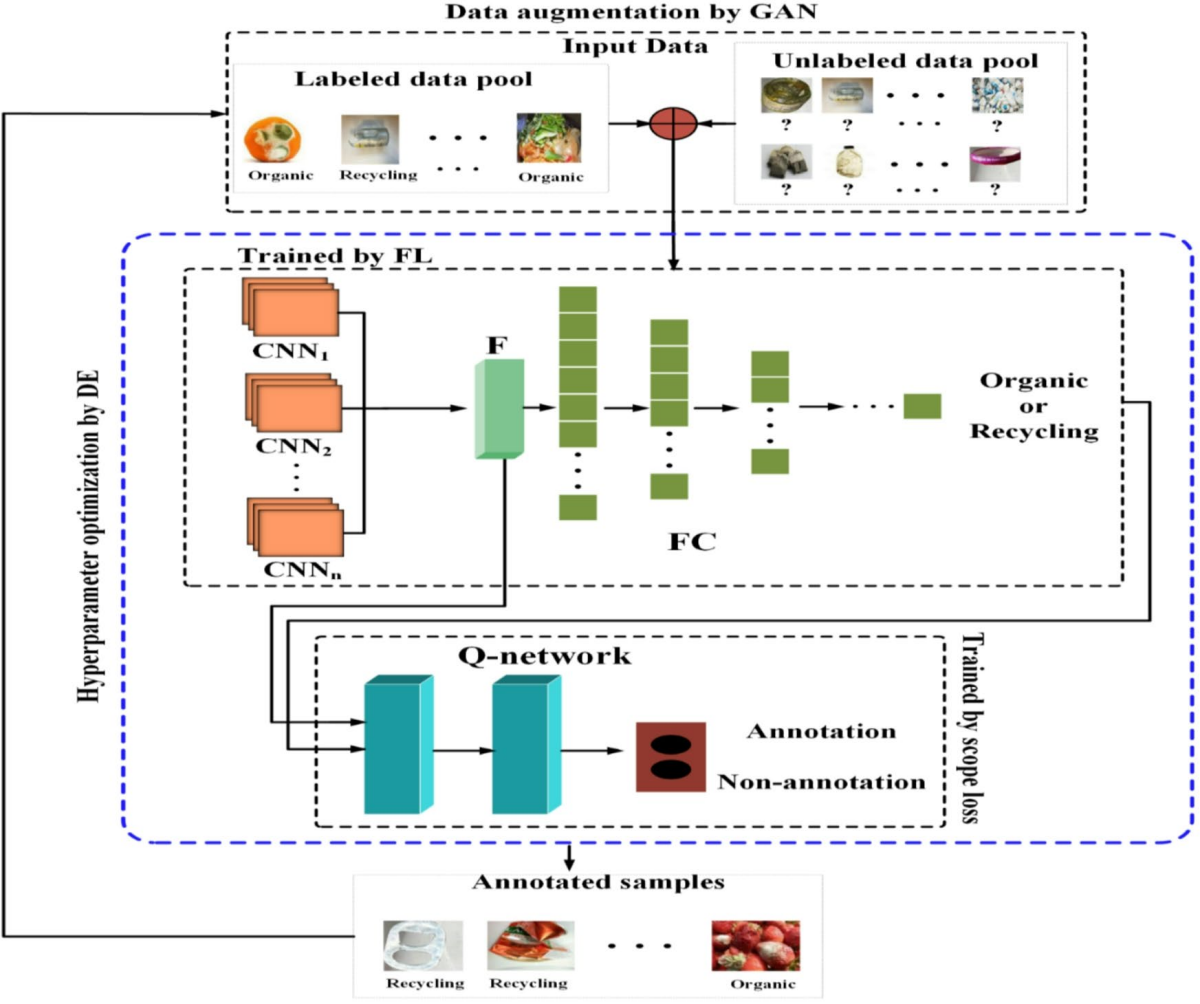
Outline of the solid waste classification framework. It includes a classifier module and a Q-network. The classifier processes image layers through several CNN branches to extract distinct feature vectors, which are then merged and passed through FC layers for final prediction. Alongside this, the Q-network uses RL to choose useful unlabeled samples and improves training with the scope loss function. The system also adds a GAN to support generalization and uses an improved DE method to tune hyperparameters.

The primary aim of the study is to come up with an AL model that applies specifically in solid waste classification. The proposed system is summarized in Fig. 1 and is divided into two major parts of a classifier and a Q-network.

The classifier is constructed with the help of several CNNs that process image information in a systematic way. These CNNs can identify diverse patterns of images along with fine-grained features in various image layers. All the branches of the CNN specialize in acquiring different types of features and come up with separate feature vectors which are the key indicators of solid waste. The resulting vectors are accumulated into an all encompassing feature matrix and a flexible layer of relaying the nets is done. This thick layer combines and filters the information removed, enabling the classifier to perceive additional impalements of the image in several perspectives and generate an integrated verdict. Lastly, these features are combined in the fully connected (FC) layers that correctly classify each image so as to produce reliable and consistent results on waste classification. Moreover, the Q-network under the active learning model uses a strategic method to choose the most beneficial unlabeled data in the training to enhance its quality. Through RL, it evaluates the possible value of each sample and ranks them in order to select the one that is likely to enhance the model performance. The system fastens learning and enhances accuracy in classification because it focuses on the high-value data. To make the training process to be effective in response to the emerging information, the Q-network evolves its sampling strategy continuously in accordance with the progress made by the classifier. An SLF is combined with the RL structure to ensure the optimal balance between exploration and exploitation. This is because the mechanism promotes exploration instead of one model being too dependent on predictable decisions. One of its advantageous qualities is that it maintains flexibility in learning and does not implement known patterns at an early stage. In such adaptive updates, the model is able to experience stronger and faster learning and thus is more resilient and effective in dynamic environments. This exploration-exploitation mixture is essential towards improving the stabilization and adaptability of training systems atop RL-uniforms.

The proposed framework offers a more efficient and flexible approach to the selection of samples in comparison to normal semi supervised learning and AL techniques. The usual methods like random sampling or uncertainty selection do not usually keep-up with the model. Conversely, the DRA plan dynamically reinvents the selection rules in use by tracking and determining the most informative elements of data at each training step. This flexibility reduces unwarranted labelling, as well as accelerating the convergence process. This also allows exploration to be further facilitated by the addition of the SLF to allow the model to have a greater number of previously unknown samples included into the training pool. Older methods would tend to be unable to balance between the exploration and exploitation of new information. This balance is well achieved when DRA combines with SLF to create a more stable, efficient and robust learning process. In order to increase the power of the model to generalize, a more sophisticated GAN is added. With this setup, the generator does not use gradients of the leader of mini-batch samples and enhances the diversity of the generated data and raises the flexibility of the model. Moreover, a hyperparameter-sensitive optimization process is used based on an optimized DE algorithm. This is an excellent DE method that uses k-means clustering to identify and introduce important clusters with new candidate solutions. In such a manner, it speeds up the process of evolutionary search and works more efficiently in conducting the exploration of the hyperparameter space.

At the beginning of training, only the labeled subset is used to initialize the classifier. The unlabeled pool is not used for direct training. Instead, its feature representations are extracted by the CNN and passed to the Q-network. The DRL agent evaluates each unlabeled sample and decides whether it should be sent for annotation. Once labeled, the sample is added to the labeled pool and the classifier is retrained. This iterative process continues until the annotation budget is consumed. Unlabeled samples are first embedded using the current classifier feature extractor before being passed to the DRL agent.

### Data augmentation

This is particularly relevant when the retrieval power of deep learning in terms of flexibility and generalization is required to be enhanced when the volume of training data is limited. Several factors are crucial in deciding the performance of models namely the diversity and the number of input samples^50^. Data augmentation is among the most effective approaches to breaking the data given, as it extends the data, which generates distorted versions of the samples given. In this way, overfitting is reduced, which enhances the resilience of the model. One of the key advantages of augmentation is that it introduces neural networks to novel variations and therefore is able to learn more generalized representations. Training on a more diverse set of altered inputs induces less dependence on individual characteristics of the original training set. It also enhances the flexibility to real-life conditions which are mostly unforeseeable and unorganized^51^. Overfitting, being one of the typical issues of a deep architecture, is when the network is over-specialized on the training data and fails to generalize on unseen samples. The issue of this is especially acute when data labelling is uncommon. This is solved through data augmentation which synthetically produces realistic and diverse variations of the original data. This procedure facilitates the styles of learning that are generalizing instead of learning the precise examples with memorization. The method is especially useful in the analysis of medical images because the process of obtaining annotated data therein is not only time-consuming but costly too. The introduction of augmented samples also assists in maintaining high diagnostic accuracy when the samples are exposed to different imaging conditions. Models were also found to be more consistent in their performance in various clinical and environmental conditions when they are exposed to changes in brightness, orientation, scale or texture^52^.

The augmentation procedure in most research applications is commonly performed offline^53^ this poses a major challenge on the issue of model scaling and flexibility. According to this strategy, all augmented samples are to be pre-generated and stored before the training takes place. To accomplish this, one needs to have several copies of each image and this improves on the storage consumption which is too high and fast consumes available disk space. Besides, there is always a necessity to load these extra files whilst training and hence computational overhead considerably raises the overall learning process^54^. Other weaknesses of offline augmentation are that it is not flexible. Once the augmented dataset has been prepared, additional changes of settings or experimentation with new transformations would usually require making the dataset again. This is a process that is time and resource consuming. Experimentation is limited by this unchanging character. It also does not allow continuous improvement of a model as far as there are new ideas of augmentations. Also, it can be redundant to use the same pre-generated samples in several epochs. Instead of encouraging diverse feature representation this will induce the network to learn cyclic forms and hence limit its generalizability and chances of overfitting^55^.

On the other hand, online augmentation is more flexible and efficient and produces modified samples in real time during training. As those variations are created when needed rather than pre-stored the method greatly reduces the storage requirements and simplifies the data manipulation. This model experiences new and unique variations during every iteration of the training process, which increases its capacity to make predictions on how it will perform outside the training set. Adaptability is also presented by this real time augmentation strategy. Various methods of transformation can be tweaked or fine-tuned as learning advances. These constant changes and encapsulations enhance the robustness of the models. They also enhance the capability of managing hidden information and enhance the overall effectiveness of training^56^.

**Fig. 2:**
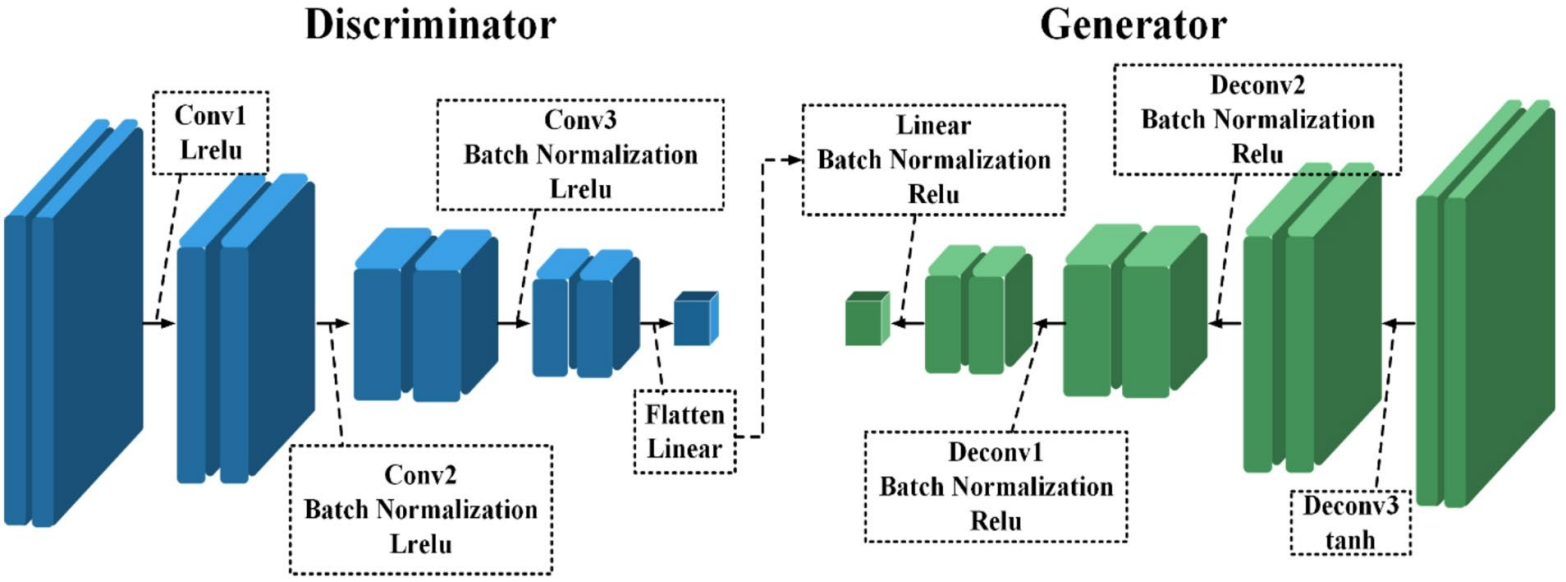
Architecture of the proposed GAN for online data augmentation.

The study presents a new real-time augmentation model that is based on a GAN system. The second numerical layer of the discriminator in the proposed model is connected with a mid-level layer of the generator. This relation allows a deeper sharing of acquired representations. Here the standard random noise vector is processed in the generator in this dual input arrangement. It also handles semantically rich features which are obtained by means of real training cases. This combination can enable the network to generate synthetic data which appears to be real. It is also a reflection of the actual distribution of authentic pictures. Figure 2 illustrates the GAN architecture used during the pre-training phase. The approach helps in the production of more diverse and high-quality data by refining diversity and fidelity. In turn, it provides more stability, adaptability, and general robustness of the model in the course of training.

The discriminator is designed in such a way that it has three consecutive convolutional layers Conv1, Conv2, and Conv3 which sequentially reduce the amount of realism in the evaluation of the input images. Conv1 in the first layer uses the 3 × 3 kernel (stride = 1, padding = 1). This is ideal in the sense that it captures small textures as well as the edge information without distorting the spatial characteristics of the input. Conv2 layer maintains identical kernel and padding coefficients but doubles the stride hence decreasing the resolution and permitting the network to scoop more abstract, mid level representations. Conv3 is akin to this configuration and is based on a 3 × 3 kernel, the stride value is 2, whereas a padding value is 1. This further compresses space information and fits lofty level semantic designs. A convolutional block is then followed by batch normalization (BN). This enhances stability of training and an accelerated convergence through regulating internal feature variance. After that, there is a leaky rectified linear (LReLU) activation used to retain small negative gradient. This avoids the inactivity of neurons and provides more efficient optimization which is needed when learning adversarially. Conv3 output is next flattened in order to produce a small feature set that allows the discriminator to tell whether an input image is natural or fake. These representations that are obtained are also provided to the generator to help direct it to produce more realistic works. This compact web composition intentionally incorporates user-redundant sizes of the kernel, structured down-sampling/ normalization and adaptive activations. The combination of these components facilitates the balanced extraction of features along with constant performance during the process of the GAN training.

The generator network comprises four deconvolutional layers, labeled Deconv1 to Deconv4. These layers progressively reconstruct detailed, high-resolution images from latent representations. After each layer, batch normalization is applied to regulate feature statistics and maintain consistency with the distributions found in real image data. In Deconv1, a 4 × 4 kernel with a stride of 2 and a padding of 1 is used to upsample feature maps while preserving spatial integrity. Deconv2 employs the same configuration to further enlarge the feature dimensions, continuing the process of structural refinement. The upsampling continues through Deconv3 and Deconv4. Each stage focuses on reconstructing finer spatial details and texture elements. For activations, ReLU is utilized in the first three layers (Deconv1–Deconv3) to introduce non-linearity and ensure efficient gradient propagation, which supports the formation of varied and realistic visual features. In contrast, the final layer, Deconv4, adopts a Tanh activation to constrain output values within the normalized range of − 1 to 1. This choice improves visual sharpness. It also prevents gradient explosion and aligns generated images with real data distributions. The generator combines hierarchical upsampling, normalization, and activation tuning in a structured way. This process produces lifelike, high-fidelity synthetic samples, thereby improving image realism and training stability.

To ensure transparency and reproducibility of the experimental framework, this section provides a detailed description of the datasets, data partitioning strategy, initial labeled configuration, and annotation budget settings employed in the active learning process.

*TrashNet Dataset*: Experiments were first conducted on the widely used TrashNet dataset, which contains 2,527 RGB images across six categories: glass, paper, cardboard, plastic, metal, and trash. The dataset reflects real-world waste conditions, including variations in lighting, background clutter, and object orientation, making it suitable for evaluating robust waste classification systems. To simulate a realistic active learning scenario with limited annotation resources, the dataset was split into training (70%), validation (15%), and test (15%) sets. Within the training set, only 10% of samples were initially labeled, forming the initial labeled pool (XLX_LXL), while the remaining 90% were treated as unlabeled data (XUX_UXU) accessible solely to the DRL-based selection agent. An annotation budget equivalent to 30% of the total training samples was imposed, ensuring that the model strategically selects the most informative samples rather than relying on full supervision. The iterative selection process continued until the predefined budget was exhausted.

*Trash Dataset*: The second benchmark, the Trash dataset, contains 3,907 images distributed into four classes: glass, paper, metal, and plastic. Compared to TrashNet, this dataset exhibits higher intra-class variation and class imbalance, making it suitable for evaluating the robustness of active learning strategies. The data were split using the same protocol (70% training, 15% validation, 15% test). Only 10% of the training samples were initially labeled, while 90% remained unlabeled and were dynamically evaluated by the Q-network during the active learning loop. A controlled annotation budget of 30% of the training data was enforced, enabling assessment of how efficiently the proposed DRL-based strategy reduces labeling requirements while maintaining classification performance.

*OrgalidWaste Dataset*: To further assess generalization, experiments were conducted on the OrgalidWaste dataset, which contains approximately 5,600 images evenly distributed across four classes: glass, metal, organic, and paper, with roughly 1,400 images per class. The same standardized split was applied (70% training, 15% validation, 15% test). Only 10% of training samples were initially labeled, while the remaining 90% formed the unlabeled candidate pool. The annotation process was constrained by a 30% labeling budget relative to the training set, ensuring that performance improvements were achieved through intelligent sample selection rather than increased labeling volume.

*Active Learning Initialization Protocol*: A unified protocol was applied across all datasets. The classifier was initially trained using only the labeled pool. Unlabeled samples were not directly used for supervision; instead, their feature representations were extracted and evaluated by the DRL-based Q-network. At each iteration, the agent selected a small batch of informative samples within the annotation budget. Newly labeled samples were added to the labeled pool, and the classifier was incrementally retrained. This process continued until the annotation budget was fully consumed. This experimental design allows a fair comparison between fully supervised and active learning configurations and highlights the labeling efficiency of the proposed framework.

**Algorithm 1:**
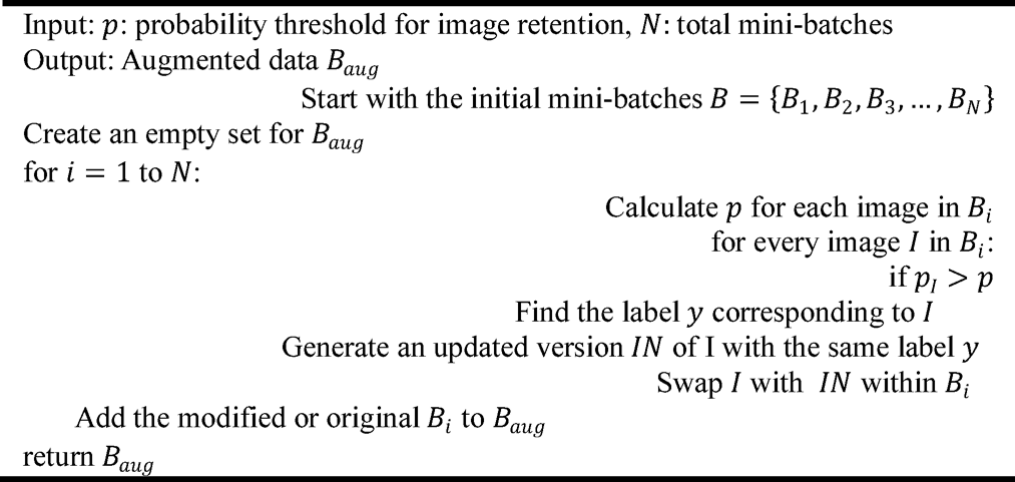
The online image augmentation procedure.

#### Loss function

GANs are composed of two primary elements: the generator (G) and the discriminator (D). The generator fabricates synthetic data instances, whereas the discriminator assesses each instance to discern if it originates from the actual dataset or is artificially produced by the generator. The objective of the discriminator is to attribute a high probability (approaching 1) to authentic samples and a low probability (approaching 0) to synthetic ones created by the generator using random noise inputs^57,58^.

The discriminator utilizes binary cross-entropy for its loss function, described as follows:

1 $$\:{L}_{D}\:\text{}\:=-\left({\mathbb{E}}_{x\:\in\:\:{p}_{data}\left(x\right)}\:\text{}\:\right[{log}D\left(x\right)]+{\mathbb{E}}_{x\:\in\:\:{p}_{z}\left(z\right)}\:\text{}\:[log(1-D(G\left(z\right)\left)\right)\left]\right)\:$$

In this formula, $\:{\mathbb{E}}_{x\:\in\:\:{p}_{data}\left(x\right)}\:\text{}\:\left[{log}D\left(x\right)\right]$ represents the expected log probability that the discriminator will accurately classify actual samples as real. Conversely, $\:{\mathbb{E}}_{x\:\in\:\:{p}_{z}\left(z\right)}\:\text{}\:\left[log\right(1-D\left(G\right(z\left)\right)\left)\right]$ indicates the likelihood that the discriminator will properly identify synthetic (fake) samples as false.

Meanwhile, the goal of the generator is to produce synthetic samples that convincingly mimic real data, deceiving the discriminator into misclassifying these as genuine. The generator also uses binary cross-entropy for its loss function, which is outlined as follows:

2 $$\:{L}_{G}\text{}\:=-{\mathbb{E}}_{x\:\in\:\:{p}_{z}\left(z\right)}\:\text{}\:\left[logD\right(G\left(z\right)\left)\right]$$

Here, $\:{\mathbb{E}}_{x\:\in\:\:{p}_{z}\left(z\right)}\:\text{}\:\left[logD\right(G\left(z\right)\left)\right]$ represents the expected value over the noise distribution of the log probability that the discriminator mistakenly identifies a synthetic sample as genuine.

In our GAN setup, the discriminator plays a central role by learning important features from the training set, and the generator relies on these features to produce new data. To increase training stability and avoid mode collapse, we add a reconstruction loss term to the generator’s objective function, as shown below:

3 $$\:{L}_{r}={E}_{x\:\in\:\:{p}_{x}}\:\left[\right|\left|G\right({D}_{F}\left(x\right))\:-\:x|\left|\right]$$

In this formula, $\:{D}_{F}$ refers to the discriminator without its final output layer. It transforms the input into a feature-based representation. The function of the generator is to rebuild the original structure of this representation.

The loss function of the generator is calculated as:

4 $$\:{L}_{G}+{\uplambda\:}\:\mathrm{*}{L}_{r}$$

Under this arrangement, the parameter λ is used as a coefficient by which the importance laid on the reconstruction loss is determined. This loss is significant in maintaining output diversity in the GAN and in solving the widespread problem of mode collapse, as with Eq. 4 stated. The mode collapse occurs when the generator continues to produce the same outputs and does not represent the total variation existing in real data. Making sure that the reconstruction is correct makes the GAN encouraged to produce those outputs that are realistic and varied, which are well-reflective of the variability of the input data. This reconstruction term can be adaptionally tuned with the λ parameter such that it can be adjusted to the properties of every dataset or application. This flexibility balances the strict reproduction of information trends and the presence of creative changes in the results produced. By doing so, the generator and the discriminator are more easily trained in conjunction, thereby eliminating any instability or inefficiency that would otherwise cause poor performance of the model. Consequently, the method prevents mode collapse. It encourages a collaborative learning strategy whereby the generator and the discriminator are constantly refined to produce quality and diverse and natural generated examples.

#### GAN training protocol and integration with active learning

To clarify the augmentation workflow, the GAN is pre-trained prior to the start of the active learning (AL) loop. Specifically, the generator and discriminator are trained using the initial labeled dataset until convergence of the adversarial objective.

After this pre-training phase, the generator parameters are frozen and no further adversarial updates are performed during the AL process.

During each AL iteration, the fixed generator is used to synthesize additional samples on-the-fly. These generated samples are incorporated only within the current mini-batch used to update the classifier. They are not permanently stored in the labeled pool and do not modify the GAN parameters.

Therefore, the augmentation process is dynamic in sample generation but static in GAN optimization. This design ensures computational efficiency, prevents instability caused by concurrent GAN retraining, and preserves sample diversity across iterations.

The GAN is trained only on the initial labeled seed set L0 before the active learning loop begins. No unlabeled data or test samples are used during GAN training. After convergence, the generator parameters are frozen and remain unchanged during active learning. At each AL iteration, the generator produces synthetic samples that are used only for temporary batch-level augmentation of classifier training and are not incorporated into the labeled pool.

**Algorithm 2:**
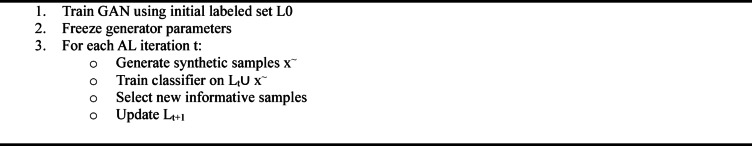
GAN-Augmented Active Learning.

### Classifier

We review an image dataset $X\:={\left\{{x}_{i}\right\}}_{i=1}^{N}$​, where $\:{x}_{i}$​ denotes the i-th image in the training set, and N represents the total number of these training images. These images are sorted into categories $\:c=\left\{{c}_{i}\right\}$, each $\:{c}_{i}\:$representing one of C distinct classes. The portion of the dataset that contains labeled images is indicated as $\:{X}_{L}={\left\{\right({x}_{{L}_{i}},{y}_{{L}_{i}}\left)\right\}}_{i=1}^{{N}_{L}}$​​, where $\:{x}_{{L}_{i}}$​​ is the i-th labeled image, $\:{y}_{{L}_{i}}$ is the corresponding class label, and NLN_LNL​ denotes the total number of labeled images. Conversely, the unlabeled images are categorized as $\:{X}_{U}={\left\{{x}_{{U}_{i}}\right\}}_{i=1}^{{N}_{U}}$​​, where $\:{x}_{{U}_{i}}$ is the i-th unlabeled image, and $\:{N}_{U}$ is their total count. The proposed framework adopts a purely value-based Deep Q-Network (DQN) algorithm. No policy-gradient, actor–critic, or PPO-based optimization is employed in the final model.

The DRL model for selecting samples operates as outlined below:

- State: At each decision juncture, the agent assesses the prevailing conditions, requiring a comprehensive capture of vital data. The state is represented by a combination of feature vectors extracted from CNNs, noted as $\:{f}_{i}$​, alongside their associated prediction scores $\:f\left({x}_{i}\right)$. These states are collectively defined as $\:S=\left\{{s}_{i}^{t}\right\}$, where $\:{s}_{i}^{t}=({f}_{i}^{t},f({x}_{i}^{t}\left)\right)$ and t indicates the specific time step.
- Action: The actions determine whether a sample requires human annotation. This decision space is depicted as $\:A=\left\{\mathrm{0,1}\right\}$. If $\:{a}_{i}^{t}=1$, it signals that $\:{x}_{i}^{t}$ should be labeled, leading to human annotation and subsequent inclusion in the labeled dataset. The active learning process terminates when all samples have been assessed, or the annotation budget is depleted.
- Reward: The reward structure is crucial for evaluating the consequences of the decisions of the agent. Contrary to conventional methods that allocate rewards at the end of a sequence, this model administers immediate feedback, directly linking each decision to its broader effects. This reward framework is bifurcated into two parts:

1. For actions resulting in annotation ($\:{a}_{i}^{t}=1$): The reward function applies a minor penalty of -0.2, noted as $\:R\left({s}_{i}^{t-1},{a}_{i}^{t}\right)=-0.2$. This penalty deters excessive annotation and conserves resources by discouraging unnecessary labeling.
2. Validation-Based Accuracy Estimation.
  To enable reliable online performance evaluation, 10% of the initial labeled pool is held out as a fixed validation subset prior to the active learning loop.
  The validation set is never used for training or sample selection.
  At iteration ttt, the model accuracy Acc_t_ is computed exclusively on this validation subset.
  This ensures that no information leakage from the unlabeled pool occurs during reward computation.
  Adaptive δ Update Rule.
  The parameter δ is dynamically adjusted according to validation accuracy:
  $${\delta _t}={\delta _0} \times (1 - Ac{c_t})$$
  where:
  ${\delta _0}$ is an initial scaling factor.
  $Ac{c_t}$ is validation accuracy at iteration t.
  Acc _t_ is computed on a fixed validation split separated from the active learning poo.
  This mechanism provides:
  Larger δ when accuracy is low → stronger exploration.
  Smaller δ when accuracy increases → stabilized exploitation.
3. For actions that forego annotation ($\:{a}_{i}^{t}=0$): The model calculates $\:{m}_{c}$, representing the centroid of the feature vector for class c. This centroid is computed based on the aggregated features of class c samples, facilitating a more measured approach to managing annotations. This reward is calculated as follows:

5 $$\:{m}_{c}=\frac{1}{{n}_{c}}\sum\:_{i\:\in\:\:{\pi\:}_{c}}{f}_{i}$$

#### Theoretical justification of the reward design

Unlike classical uncertainty-based active learning, the proposed reward mechanism discourages redundant labeling of samples located near stable class centroids. Samples close to centroids are already well represented in the feature space and provide limited marginal information gain.

Therefore:

- Samples near decision boundaries receive higher incentive for labeling.
- Samples near centroids receive reduced labeling priority.

This design improves label efficiency while maintaining informativeness, which is further validated through ablation analysis in Section “Empirical evaluation”.

In this context, $\:{n}_{c}={|\pi\:}_{c}|$ denotes the number of samples associated with class c. These class centroids are then employed to compute the entropy loss function, which is directed toward the unlabeled samples.

6 $$\:L\:\left({x}_{{U}_{i}},\:c\right)=-\sum\:_{j=1}^{C}\frac{{e}^{-{\left({f}_{{U}_{i}}-{m}_{{c}_{j}}\right)}^{2}}}{\sum\:_{j=1}^{C}{e}^{-{\left({f}_{{U}_{i}}-{m}_{{c}_{j}}\right)}^{2}}}{log}\frac{{e}^{-{\left({f}_{{U}_{i}}-{m}_{{c}_{j}}\right)}^{2}}}{\sum\:_{j=1}^{C}{e}^{-{\left({f}_{{U}_{i}}-{m}_{{c}_{j}}\right)}^{2}}}$$

For each instance, $\:{f}_{{U}_{i}}$​​ represents the feature vector of the i-th unlabeled image extracted by the CNNs. Subsequently, the reward function for when $\:{a}_{i}^{t}=0$ is calculated as follows:

7 $$\:R\left({s}_{i}^{t-1},{a}_{i}^{t}\right)=\left\{\begin{array}{c}1,\:\:\:L\:\left({x}_{{U}_{i}},\:c\right)<\delta\:\\\:-1,\:\:L\:\left({x}_{{U}_{i}},\:c\right)>\delta\:\end{array}\right.$$

In this framework, $\:L\:\left({x}_{{U}_{i}},\:c\right)$ quantifies the entropy loss for each unlabeled image $\:L\:\left({x}_{{U}_{i}},\:c\right)$, while δ is a variable parameter that adjusts dynamically in response to the increasing accuracy of the model. This reward mechanism incentivizes the model to prioritize annotations for samples with lower confidence, evolving beyond traditional uncertainty-based selection methods. In this adaptive approach, the criteria for selection are dynamically learned rather than statically predefined. Early in the training phase, δ is maintained at a lower setting to mirror the initial unreliability of the feature extraction. As the training advances and the accuracy of the model enhances, δ is incrementally increased, reflecting improved reliability in the feature extraction process. This adaptive strategy ensures that as the accuracy of the model matures, it relies more on its refined assessments, seeking annotations primarily for samples crucial for ongoing improvement. This method maximizes the efficiency of labeling resources, ensuring they are utilized optimally as the model progresses.

In our model, we use a collection of CNNs that are used in feature extraction. All CNNs have five 2D convolutional layers, successively reducing to a 128, 8 size. This structure narrows the feature detection in the images. Each layer is fitted with 2 × 2 kernels and a stride of 3 and 4 paddings which efficiently strikes the features extraction speed and accuracy. After every convolutional step, there exists a 2 × 2 max-pooling layer that will reduce the spatial size of the feature maps but leave the most important features, thereby, increasing the processing speed. The result of all CNNs is the formation of vector F as shown in Fig. 1. This is the vector that divides into two paths the Q-network and the classification pathways. Q-network, the architecture that directs the decision-making incorporates two completely connected layers. The classification pathway takes the softmax-normalized output of the FC layers, f (x i ), and feeds it into the classification pathway, which produces an indicator of potential actions, a i = 0 or a i = 1, and the reinforcement learning agent picks the option with a higher score on the Q-network.

In order to learn the classifier, we apply the use of focal loss (FL)^56^ to overcome the problem of imbalance in the classes. FL is an adaptive modification of the standard cross-entropy loss. This term causes the focus to be more on harder and less predicted samples. Consequently, on this reason the model does not over-fit to the dominant class. The strategy enhances the classifier as it makes the classifier more receptive to minority classes. FL assists more equitable learning and enhances the performance on those datasets in which classes are disproportionate by emphasizing more on troublesome cases.

**Fig. 3:**
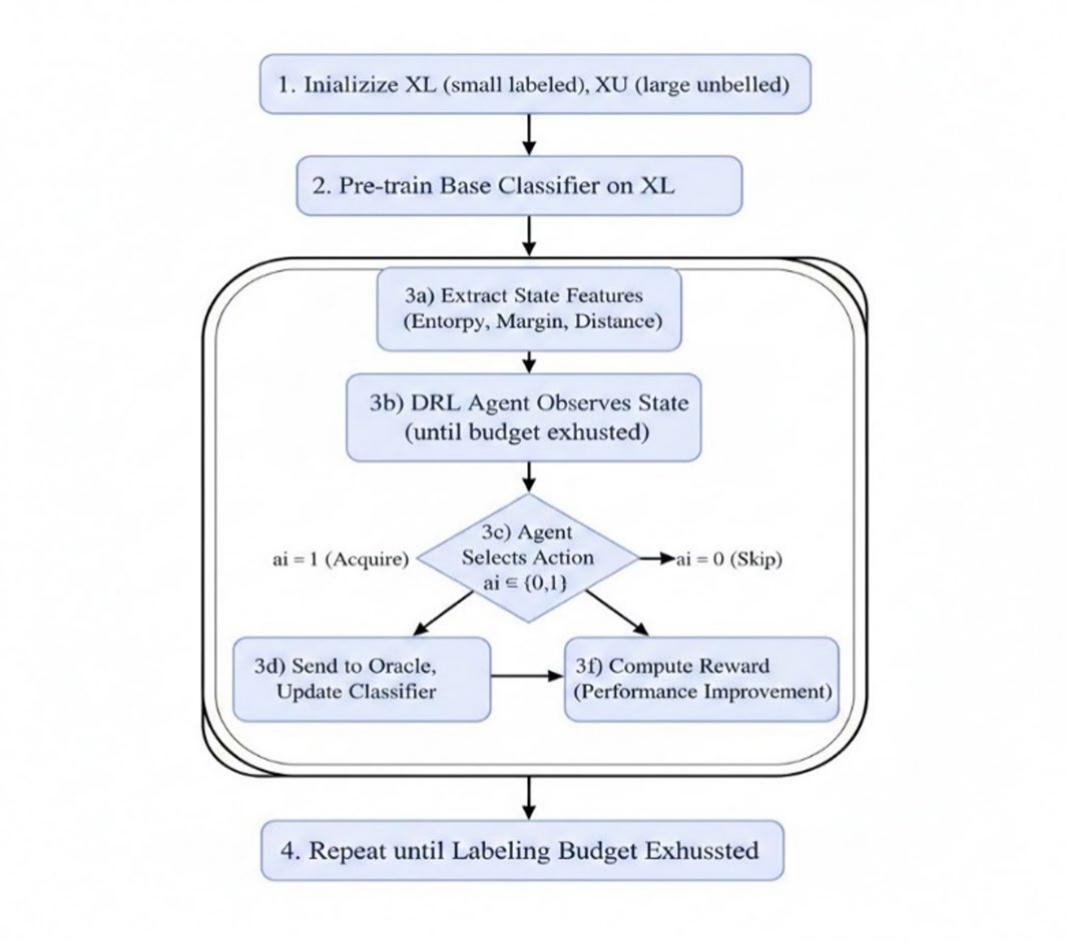
The architectural workflow of the proposed DRL-based active learning framework, illustrating the closed-loop interaction between the base classifier (environment) and the DRL agent.

The training process of the Q-network begins by forming a tuple $\:({s}_{i}^{t},{a}_{i}^{t},{r}_{i}^{t},{s}_{i}^{t+1})$ that captures a single transition in the agent’s interaction history. This tuple reflects the outcome where the agent takes action $\:{a}_{i}^{t}$ in state $\:{s}_{i}^{t}$, receives reward $\:{r}_{i}^{t}$, and shifts to the next state $\:{s}_{i}^{t+1}$. To support effective learning of the data selection method, a scope loss function is integrated into the DRL framework (Fig. 3).

To clarify the integration of the DRL agent with the classifier’s training, the overall process is structured as follows:

1. Initialization: Initialize a small labeled set X_L_ and a large unlabeled pool X_u_, then pre-train the base classifier on X_L_.
2. AL Iteration: At each iteration:
  - State Extraction: Extract features (entropy, margin, distance to centroid) from the classifier.
  - Observation: The DRL agent observes the state.
  - Action: The agent selects action a_i_ ∈ {0,1}.
  - Labeling: If a_i_ = 1, the sample is labeled by the oracle and added to X_L_.
  - Model Update: The classifier is incrementally updated.
  - Reward: Reward is computed based on performance improvement.
3. Termination: Repeat until the labeling budget is exhausted.

**Algorithm 3:**
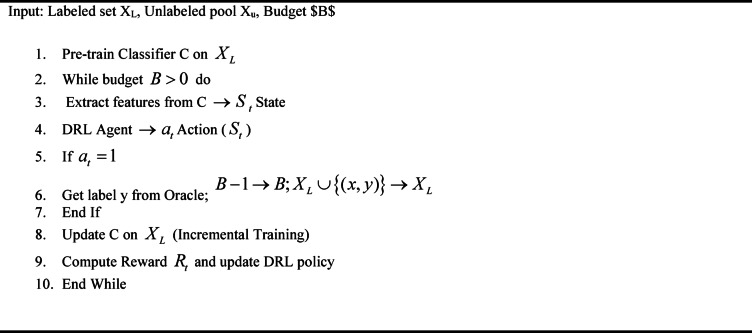
The Unified DRL-based Active Learning Framework.

#### SLF

Proper balance between exploration and exploitation is important in RL. The way agents learn is through the interaction with the environment and their actions have a direct influence on the way learning takes place. They have to trial a number of activities to determine which ones can provide beneficial results and also utilize available knowledge to optimize reward. At the early stages of learning, agents usually focus on exploration in order to learn more about the environment. As learning moves forward they move more onto exploitation in order to polish the decision making process. Exploration is sufficient to enable more powerful exploitation strategies and excess exploitation can inhibit learning and achieve poorer overall performance^57^.

Policy loss, entropy, and FL are included in the SLF, and are meant to solve three problems of RL, namely: efficient use of rewards signals, premature convergence, and action selection imbalance. Policy loss term informs the agent to realize its actions based on the advantage estimate, and maximize expected rewards. The entropy component facilitates exploration since it disincentivizes early convergence to deterministic policies, having even more resilient policies. FL, which was originally proposed in supervised learning, is used to address the issue of imbalance in classes by using dynamic weighting. It backfills the influence of overconfident behaviors and more highly values uncertain ones and this limits overfitting and bias when selecting the action.

In theory, this composite loss will serve as a gradient control. It not only varies the magnitude and direction of policy changes depending on the confidence of action but also the perceived benefit. This is the mechanism that agrees with the initial objective of keeping a balance of exploration and exploitation in policy gradient techniques. Combining these three components, the SLF functions as an integrated surrogate loss which will retain the merits of both of them and will counteract the weaknesses of each.

The SLF is more trainable as compared to normal general RL loss expression, like the simple policy gradient or policy-entropy hybrids. It is also more efficient in exploration and distributes actions. It is also substantiated by experimental evidence that this framework hastens convergence as well as the capacity of the model to generalize to novel learning conditions.

**Algorithm 4:**
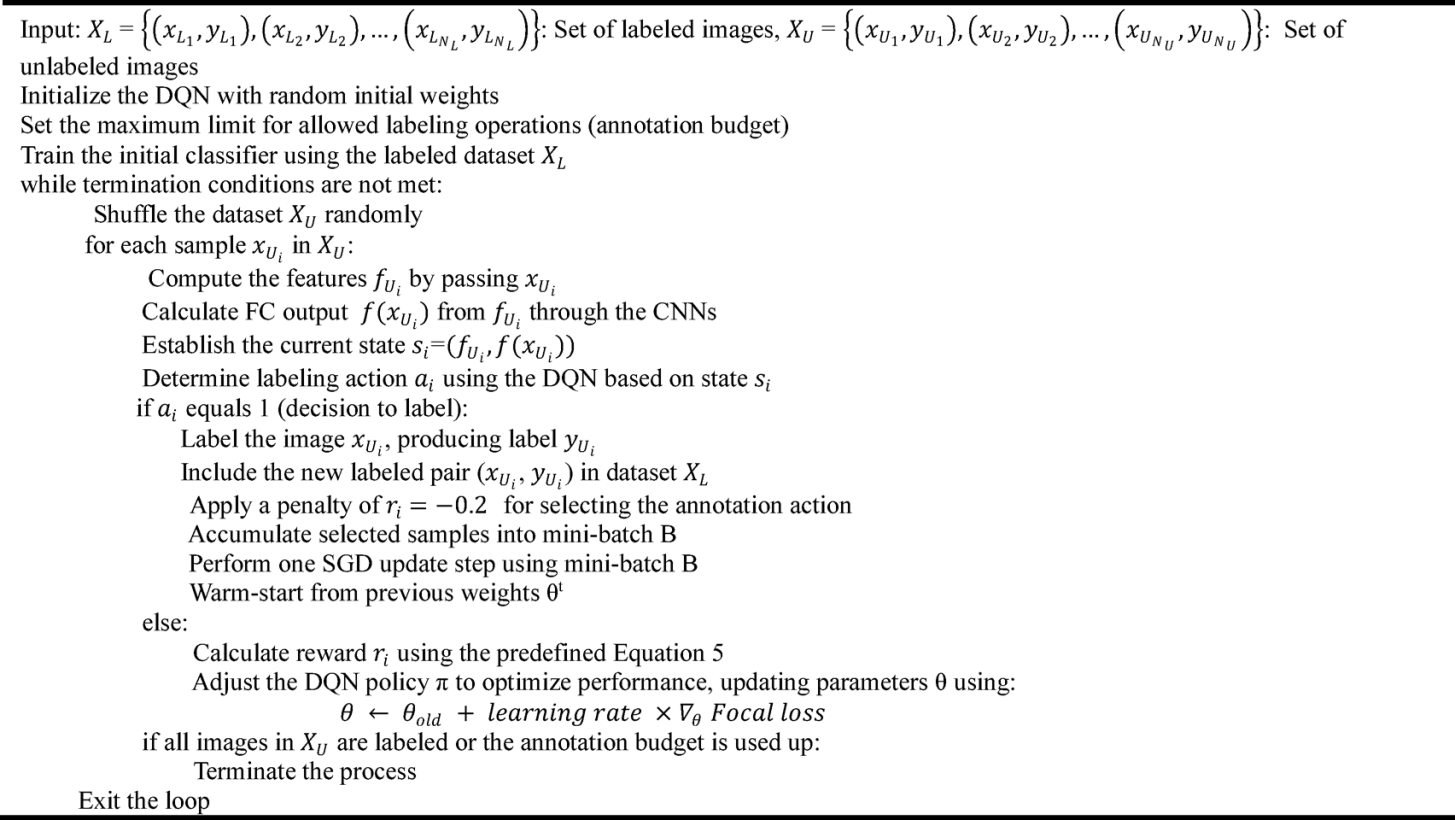
Pseudo-code of the active learning algorithm.

To ensure computational efficiency, the classifier is not retrained from scratch after each labeled sample. Instead, newly selected samples within one AL iteration are accumulated into a mini-batch of size B = 32. The model parameters are warm-started from the previous iteration and updated using a single stochastic gradient descent (SGD) step. No full-epoch retraining is performed. This incremental strategy significantly reduces computational overhead and enables practical scalability. The AL framework pseudocode is displayed in Algorithm 2. It provides a step by step process of labeling the unlabeled images effectively and optimally improving the performance of the classifier. The two datasets included in the framework are the labelled set X L and the unlabelled set X U, which contains a pair of images and labels and does not have any labels respectively. To start with, the deep Q-Network (DQN) is randomly-initialized and annotation budget is specified to limit the amount of time spent on manual labeling. The classifier is put through some initial training on X L to allow a baseline model. The other AL cycle recursively working on X until the annotation limit or all of the samples in X has been processed. X U is randomly shuffled in every iteration. The system computes the CNNs features of each unlabeled image x (U i ) and calculates the FC representation f(x(U i ) of the image. The current state s i is provided by this representation, after which the DQN will calculate the result to indicate whether the sample should be labeled or not. When the DQN decides to label the image and do this, then it is annotated, appended to X_L and a small penalty is imposed to indicate the cost of labeling the image. This probation dataset is then reused to update the classifier. On the contrary, when the picture gets not chosen to be labelled, a reward is allocated.

**Table 2:** Overview of hyperparameters fine-tuned in the study.

Hyperparameter	Range	Best set value		
		RL	Generator	Discriminator
Batch size	16 to 1024	73	46	56
Learning rate	[0.0001 to 0.1]	0.0015	0.0085	0.002
Epoch	32 to 2048	312	175	196
Activation function	{ReLU, Leaky ReLU, Tanh, Sigmoid}	ReLU	ReLU	ReLU
Dropout rate	[0–1]	0.15	0.29	0.38
λ	[0–1]	-	0.21	-
Noise size	16 to 512	-	62	-
Number layer in MLP	1 to 8	3	-	-
Number of stacked CNNs	1 to 8	3	-	-
$\:\gamma\:$	[0–1]	0.018	-	-

### Hyperparameter optimization

Deep learning and DRL require the process of tuning hyperparameters, which greatly increases the level of learning process efficiency and overall model performance. Well-applied hyperparameters may enable faster convergence of training and higher accuracy and stop excessive overfitting of the model. This in deep learning entails the adjustment of the learning rate, number of epochs, and batch size which has a direct implication on the effectiveness of network-learning it based on the data^58^. In the case of DRA, the best hyperparameters are used so that there is a balance between exploration and exploitation; this is paramount in ensuring the agent can learn the effective policies without opting to get him/herself stuck on the suboptimal states. A well-tuned model can result in stronger and more general comprehensible models that can also work well on the unknown data in different tasks^59^.

Table 2 outlines the hyperparameters adjusted during our investigation, with ranges established by precedents in prior solid waste classification studies.

#### Random Key

The paper will use the Random Key approach^60^ to optimize hyperparameters due to its effectiveness in managing the highly dimensional nature of hyperparameter space of a deep learning model. Random Key approach When dealing with hyperparameter tuning, the approach of random key can be used to transform it into a combinatorial optimization problem, which can be solved with a variety of metaheuristic algorithms. Such an approach is also flexible to be able to smooth continuous and categorical hyperparameters by representing them as numeric keys, which are subsequently decoded in the course of optimization. This kind of representation permits the approach to search through an extensive variety of parameter settings, which increases the possibility to find the most effective hyperparameter settings. Also, the Random Key method is less susceptible to local optima compared to other strategies, such as grid or random search, and hence is more applicable to high dimensional spaces and more complex models where other techniques fail.

**Fig. 4:**
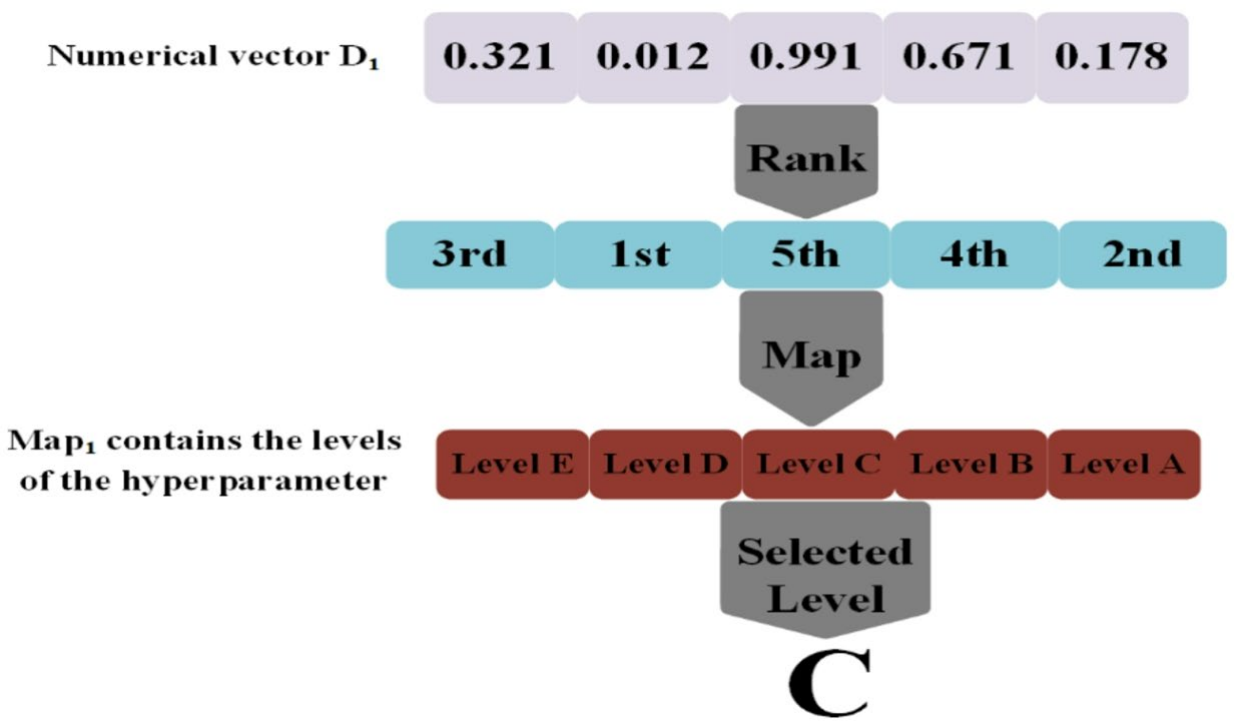
Illustration of the Random Key approach with $\:{D}_{c}=5$.

The Random Key approach acts as an encoding method that uses $\:T$ numerical vectors, each containing $\:D$ features. These vectors are labeled $\:{p}_{1},\:{p}_{2},\:...,\:{p}_{T}$ and represent possible model configurations. A mapping process, called the random key, connects each vector to a set of model hyperparameters. To adjust $\:C$ hyperparameters, the method links each one to $\:{D}_{c}$ positions in the vector. For continuous parameters, $\:{D}_{c}\:$equals one. The total vector size, $\:D$, is determined by summing all $\:{D}_{c}\:$values from $\:c\:=\:1$ to $\:C$. Each vector $\:{p}_{i}\:$is divided into $\:C$ segments, with every segment holding $\:{D}_{c}$ values. These segments point to potential settings for each hyperparameter. Continuous values are scaled to fall within the allowed range. For categorical parameters, the $\:{D}_{c}$ part of $\:{p}_{i}$ is converted into a list named $\:{MAP}_{c}$, which contains all valid choices for the $\:{c}^{th}$ parameter. This is done by ranking the $\:{D}_{c}$ entries and selecting the highest one as an index to choose a value from $\:{MAP}_{c}$. This method allows evolutionary techniques like crossover, mutation, and selection to operate on numeric vectors. It also ensures that the modified vectors can be decoded back into meaningful and valid hyperparameter choices. Figure 4 depicts this approach for $\:{D}_{c}$=5.

#### DE

To gain a better output of the Random Key technique, its functionality is combined with the DE algorithm since the algorithm is strong in dealing with global optimization problems. DE assists in enlarging the search procedure by implementing the mutation and crossover which produce a multiplicity of potential solutions. This diversity enhances the capability of the algorithm to cover the parameter space and not to fall into local optima. DE with Random Key encoding model is used to formulate the search of complex hyperparameter configurations more effectively. The simplicity of the DE system is compatible with the Random Key system. It works out candidate solutions by updating on the basis of fitness values which results in better tuning results. This hybrid method has the benefit of increasing accuracy besides accelerating convergence. It can be applied in particular when tuning deep learning models, whose search space is large and performance can be tremendously affected by parameter modifications.

DE technique has three major processes namely: mutation, crossover and selection. It begins with mutation that is critical to provide new variations to the population. At this step, the algorithm alters the existing solutions by blending elements of other members of the group. It normally modifies a solution of interest by incorporating a scaled difference between 2 other candidates and generates a new one. In such a way, diversity of the population is retained and new possibilities are explored. In such a way, the algorithm will not be satisfied with the average performance, but it will be striving to achieve more successful results. The general key to the performance of DE is the ability to produce a wide pool of varied solutions to the problem, as this is crucial towards achieving the best possible solution.

In the DE algorithm, the mutation phase produces a new vector by employing the following technique:

8 $$\:{\overrightarrow{v}}_{i,g}=\:{\overrightarrow{x}}_{{r}_{1},g}+F\:({\overrightarrow{x}}_{{r}_{2},g}-\:{\overrightarrow{x}}_{{r}_{3},g})$$

In this context, $\:{\overrightarrow{x}}_{{r}_{1},g}$, $\:{\overrightarrow{x}}_{{r}_{2},g}\:$, and $\:{\overrightarrow{x}}_{{r}_{3}}$ represent three different candidate vectors chosen at random from the present population. The factor. The parameter $\:F$ acts as a scaling term that decides how strongly the difference between two vectors affects the generation of a new one. When the mutation step ends, the algorithm moves to the crossover stage. In this stage, elements from the mutated vector are mixed with those of the target vector. This mixing is usually performed through the binomial crossover method:

9 $$\:{u}_{i,j,g}=\left\{\begin{array}{c}{v}_{i,j,g}\:if\:rand\left(0,\:1\right)\le\:\:CR\:or\:j\:={j}_{rand}\\\:{x}_{i,j,g}\:\:\:\:\:\:\:\:\:\:\:\:\:\:\:\:\:\:\:\:\:\:\:\:\:\:\:\:\:\:\:\:\:\:\:\:\:\:\:\:\:\:\:\:otherwise\end{array}\right.$$

where $\:CR$ refers to the crossover rate, while $\:{j}_{rand}$ is a randomly selected index from $\:\{1,\:2,\:\dots\:,\:D\}$, where $\:D$ denotes the dimension of the candidate solution. The final step is the selection stage. In this step, the trial vector produced by crossover is evaluated against the original target vector. The algorithm keeps whichever option performs better. This stage is essential because it guides the population toward stronger candidates and ensures that only improved solutions continue to the next iteration.

**Fig. 5:**
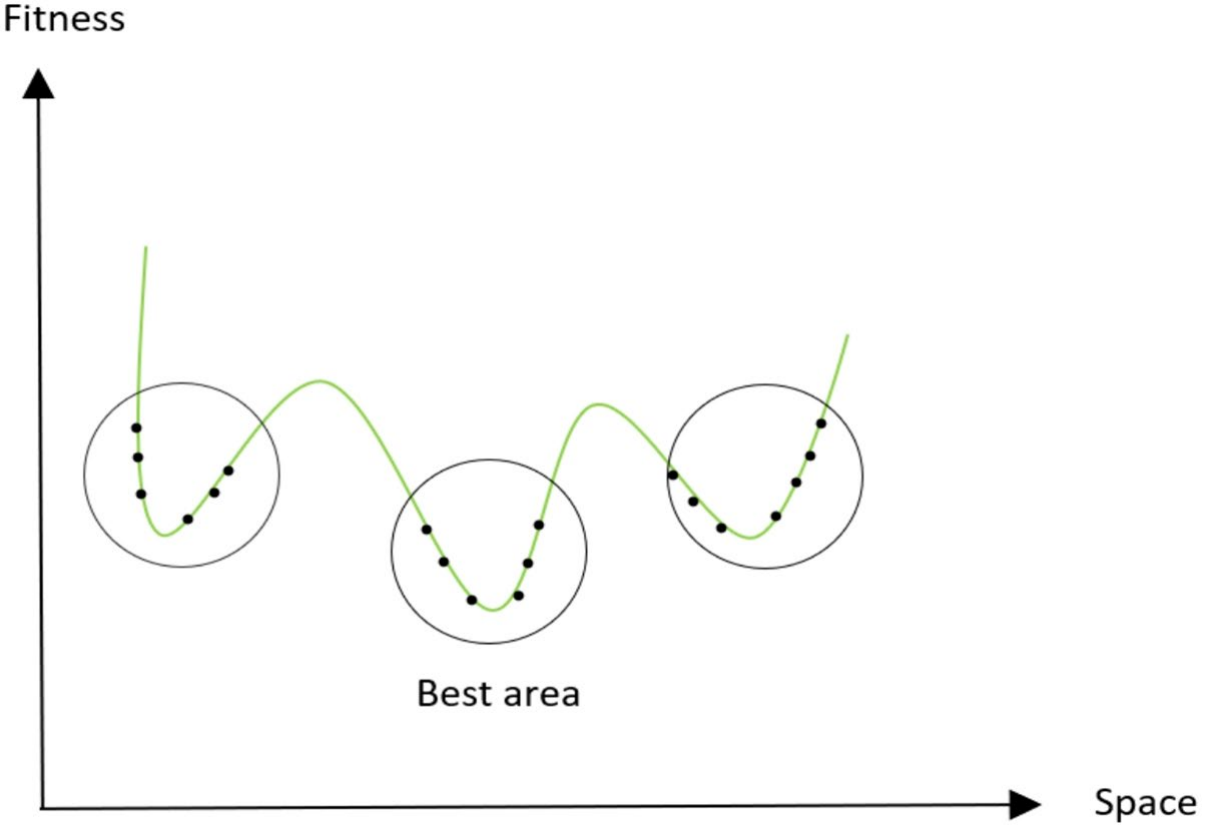
Depiction of the mutation technique of the advanced DE algorithm, which employs k-means clustering to segment 18 prospective solutions into three distinct groups.

A modified DE algorithm includes a novel mutation method based on recent findings^59^. It starts by using k-means clustering to split the population into different groups across the search space. The value of k is randomly picked between 2 and $\:\sqrt{N}$. Once clustering is done, the cluster with the smallest average objective value is chosen for deeper search. Figure 5 presents a case with 18 candidate solutions divided into three separate clusters.

We introduce a novel mutation operator inspired by clustering, defined as follows:

10 $$\:\overrightarrow{{{v}^{clu}}_{i}}=\:{\overrightarrow{win}}_{g}+F\:({\overrightarrow{x}}_{{r}_{1},g}-\:{\overrightarrow{x}}_{{r}_{2},g})\:\:\:\:\:$$

In this setup, $\:{\overrightarrow{win}}_{g}$ refers to the best-performing candidate inside a selected cluster that shows strong potential. It is important to note that this vector may not be the top solution in the full population. The clustering-based mutation process is carried out for $\:M$ iterations. After these iterations, the population is updated through several key steps defined in the generic population‑based algorithm (GPBA)^60^:

- Selection: The k-means clustering process begins by picking $\:k$ initial centroids randomly from the current candidate solutions.
- Generation: A set of $\:M$ new candidates is produced using mutation operations. These newly generated individuals are referred to as $\:{v}^{clu}$.
- Replacement: $\:M$ solutions are randomly drawn from the existing population to form a group named $\:B$.
- Update: The best $\:M$ individuals are selected from the combined set of $\:{v}^{clu}$ and $\:B$. These top-performing solutions make up a new group, $\:B^{\prime\:}$. The population is then updated by merging the remaining individuals from $\:(P-B)$ with $\:B{^\prime\:}$.

The procedural steps for this refined DE algorithm are detailed in Algorithm 5.

**Algorithm 5:**
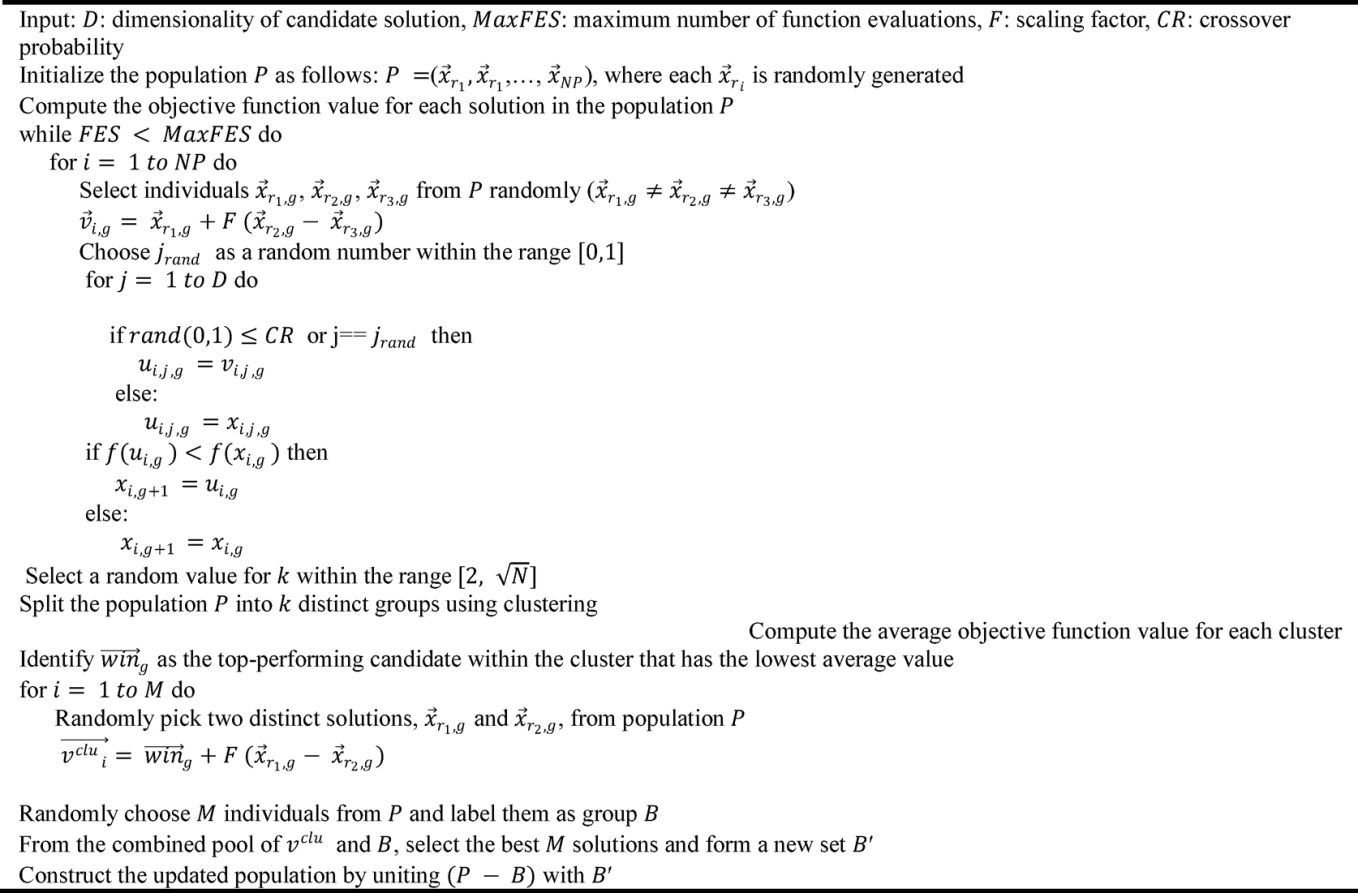
Pseudo-code of the proposed DE algorithm.

### Computational complexity analysis

The computational cost of the proposed framework consists of:

1. Forward pass for state feature extraction.
2. Q-network inference.
3. Mini-batch SGD update.

Since the classifier is incrementally fine-tuned using a single SGD step per iteration, the complexity per AL iteration is:

$${\mathrm{O}}({\mathrm{B}} \times {\mathrm{d}})$$

where B is the mini-batch size and d is the model parameter dimension. Therefore, the overall runtime scales linearly with the annotation budget and does not involve repeated full retraining. The computational cost of GAN training is incurred only once prior to the AL loop and does not affect the per-iteration complexity of the proposed framework.

### Selective gradient omission (SGO) mechanism

To enhance GAN training stability and prevent noisy gradient oscillations, we introduce a Selective Gradient Omission (SGO) mechanism applied during generator updates.

Let the standard GAN generator loss be:

$${\varsigma _G}={E_{z\sim p(z)}}\left[ {\log (1 - D(G(z))} \right]$$

${\theta _G}$ is the gradient.

$$g={\nabla _{{\theta _G}}}{\varsigma _G}$$

Instead of applying the full gradient vector g, we introduce a masking operation:

  

$$ g_{i}^{{SGO}} = \left\{ {\begin{array}{*{20}c} {g_{i} } & {if\left| {g_{i} } \right| \le \tau } \\ 0 & {otherwise} \\ \end{array} } \right. $$

where: ${g_i}$ is the i-th component of the gradient vector g.$\tau$. is an adaptive threshold.

Adaptive Threshold Definition.

The threshold is computed dynamically using gradient statistics:

  

$$\tau ={\mu _g}+\lambda {\sigma _g}$$

where: moving average of gradient magnitudes = ${\mu _g}$, standard deviation of gradient magnitudes = ${\sigma _g}$, scaling hyperparameter controlling omission aggressiveness = $\lambda$.

The moving averages are updated as:

$$\mu _{g}^{{(t)}}=\beta \mu _{g}^{{(t - 1)}}+(1 - \beta )mean(\left| g \right|)$$

$$\sigma _{g}^{{(t)}}=\beta \sigma _{g}^{{(t - 1)}}+(1 - \beta )std(\left| g \right|)\,$$

- $\beta \in \left[ {0,1} \right]$ with momentum factor.

The threshold is computed dynamically using gradient statistics:

Generator Update with SGO.

  

$$\theta _{G}^{{(t+1)}}=\theta _{G}^{{(t)}} - \eta {g_{SGO}}$$

where $\eta$ is the learning rate.

**Algorithm 6:**
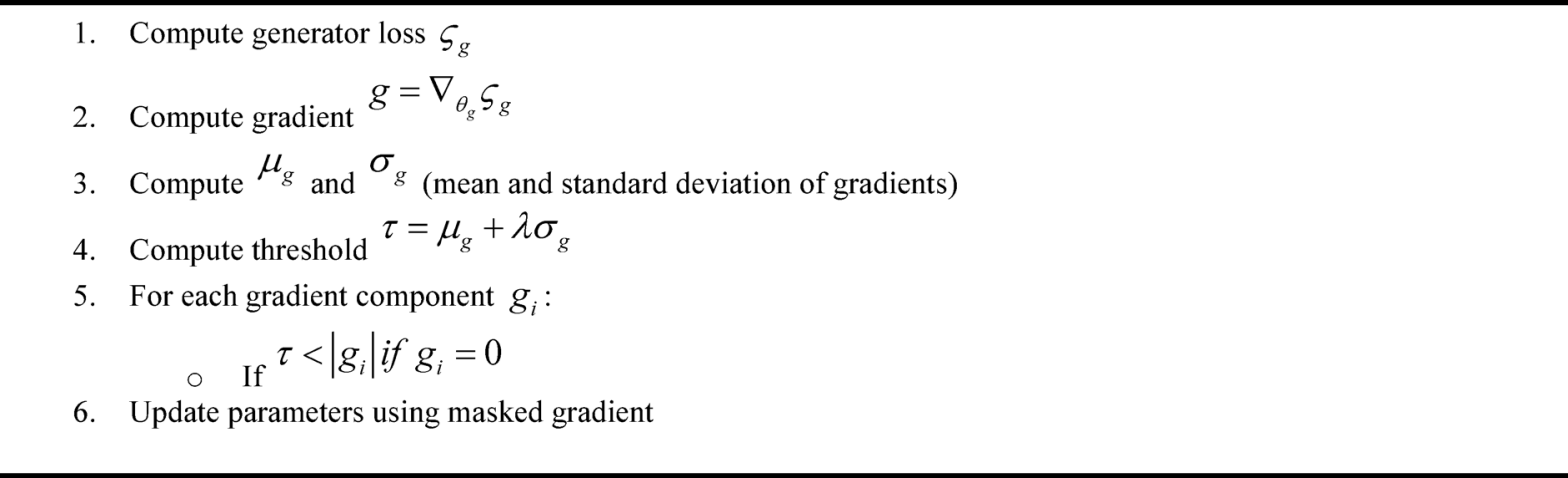
Generator Update with Selective Gradient Omission.

The proposed SGO mechanism acts as a statistically driven gradient regularizer that enhances GAN stability without introducing additional network parameters.

### Overall training procedure and system integration

To ensure the stable convergence of the interacting modules, the training process is organized into five sequential stages. This modular approach ensures that the generative and value-based reinforcement learning components do not interfere with each other’s gradient updates.

Stage 1: GAN Pretraining The GAN is first trained independently on the entire dataset to capture the underlying data distribution. After 100 epochs, the generator is frozen and used to produce a synthetic augmentation set X_GAN_ to handle data scarcity.

Stage 2: Hyperparameter Optimization (EDE) Key parameters—including the learning rate, batch size, and the gradient omission threshold $\:\partial\:$—are optimized using Enhanced Differential Evolution (EDE). The fitness function is defined as the validation accuracy on the initial labeled subset $X_{L}^{0}$.

Stage 3: Initial Classifier Training The multi-branch CNN classifier is trained for $N$ epochs using the combined set $$X_{L}^{0} \cup {X_{GAN}}$$. This provides a warm-start for the feature extractor, which is essential for the DQN agent to receive informative state representations.

Stage 4: DQN Agent Initialization The Deep Q-Network (DQN) agent is initialized. The state s_t_ is constructed from CNN feature embeddings and uncertainty metrics. The experience replay buffer is initialized to store transitions for value-based updates.

Stage 5: Iterative Active Learning Loop In each iteration, the DQN agent selects the most informative samples to be labeled. To maintain computational efficiency, the CNN is not retrained from scratch; instead, it undergoes incremental fine-tuning (1–3 epochs) using the updated labeled pool. Simultaneously, the DQN’s Q-values are updated using the Bellman equation based on the rewards received.

**Algorithm 7:**
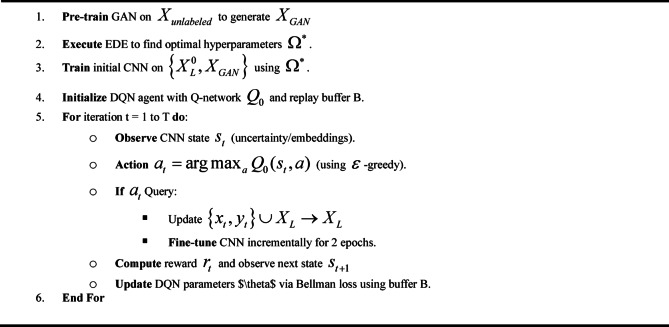
Comprehensive System Training.

## Empirical evaluation

The section begins with an in-depth dataset analysis, elucidating its characteristics and relevance to our study. Subsequently, it elaborates on the metrics used, delineating the principal benchmarks and evaluation standards for gauging the efficacy of our model. The segment concludes with the presentation of experimental outcomes, highlighting essential findings and their implications for our research objectives.

**Table 3:** Detailed specifications of the TrashNet dataset.

Class	Detail	Total data
Cardboard	Torn cardboard, card board boxes	393
Glass	Jar, broken bottles, glass, bottles	491
Metal	Can, jar lid, smashed can	400
Paper	Envelope, paper, page of magazines, newspaper	584
Plastic	Bottles, box, milk bottle	482
Trash	Different types of packets such as, chips packets, chocolate packets, sauce packets	127

**Table 4:** Detailed specifications of the OrgalidWaste dataset.

Class	Detail	Total data
Glass	Broken bottles, drinking glass, broken glass, jar	1415
Metal	Coin, aluminium foil, spoon and fork, smashed can, lid, key, cooking pot	1413
Organic	Peel, eggshell, different rotten vegetables and fruits, tea bag, meat	1435
Paper	Packets, smashed bottle, spoon, box, bottle cap, water pot, milk Jar	1420

### Datasets

This study utilized two well-established datasets to validate the effectiveness of the proposed model:

- TrashNet^61^: This dataset is accessible on platforms such as Kaggle and GitHub. It comprises a comprehensive collection of waste images categorized into six classes: glass, metal, plastic, cardboard, paper, and trash, totaling 82 MB in JPG format. The dataset features various items, including glass bottles, metal cans, plastic containers, cardboard pieces, and paper products. Table 3 presents detailed information about this dataset.
- Trash^62^: This dataset is hosted on Kaggle and encompasses 3907 images, with 136 in PNG format and the remainder in JPG, filling a total of 92 MB. It is segmented into four categories: glass, paper, metal, and plastics. Each category is specifically tailored to include relevant items such as broken glass, plastic bags, metal lids, and discarded paper products. More detailed attributes of this dataset can be found in Table 4.

To assess the generalizability of the model, we employ the OrgalidWaste dataset^63^. The name OrgalidWaste originates from combining the terms organic and solid. This dataset comprises four categories: one organic and three solid types (glass, metal, and plastic). It encompasses approximately 5600 images, totaling 93 MB. Detailed specifics of this dataset are presented in Table 5. All images are stored in JPG format. The dataset is meticulously balanced, each class containing roughly 1400 images, ensuring an equitable representation across different categories.

### Metrics

There are four major evaluation metrics applied in this study, which include accuracy, F-measure, G-means, and area under the curve (AUC), to comprehensively assess the effectiveness of the model. All measures provide alternative insights into performance as it should be fully assessed. The measure of accuracy is an indication of the number of overall predictions that were accurate. It is the proportion of true positive and true negative outcomes of all cases. The metric can be useful when the classes of the dataset are equal and represents a fast picture of overall model behaviour. F-measure or F1 score is used since it is a harmonious mean of precision and recall; it is the harmonic mean between the precision and the recall value. This measure is necessary whereby the data classes are uneven. It handles both the cases of positive identification in the model and prevents the number of false positive prediction. The reason why G-means is selected is that the geometrical mean of recall and specificity is calculated. It assists in testing the model in balanced treatment of the good and bad classes. Compared to accuracy, G-means provides a more even-handed interpretation particularly where the proportions of classes are uneven yet it also checks that performance is as good across the groups. Finally, AUC is added to demonstrate the ability of the model to discriminate the classes in various levels of threshold. This measure is typical of binary classification. It is independent of scale and particular threshold decisions, and therefore, it is a robust factor of how well the model can rank predictions.

The formulas for accuracy, F-measure, and G-means metrics are provided below^64^:

11 $$\:Accuracy=\frac{True\:Positives\:\left(TP\right)+True\:Negatives\:\left(TN\right)}{Total\:observations}\:\:\:\:$$

12 $$\:F-Measure\:\left(F1\:Score\right)=2\times\:\frac{Precision\times\:Recall}{Precision+Recall}\:\:\:\:$$

13 $$\:G-means=\:\sqrt{Sensitivity\times\:Specificity}\:\:$$

where

14 $$\:Precision=\frac{TP}{TP+False\:Positives\:\left(FP\right)}\:\:\:\:$$

15 $$\:Recall=Sensitivity=\frac{TP}{TP+False\:Negatives\:\left(FN\right)}\:\:\:\:$$

16 $$\:Specificity=\frac{TN}{TN+FP}$$

### Results

The experiment was done on a 64-bit United States of windows operating system with strong computational power to service the intensive requirements of deep learning models. Precisely we used Intel i9 processor and NVIDIA RTX 3080 graphics card, which gave us the required computing power and graphics processing ability. Our model structures were based on tensorflow and keras because of their strength to handle massive image data and vast neural network libraries to create and train the model. The programming language Python was selected due to versatility and the prevalence of scientific libraries of computing (NumPY and SciPy) necessary to manipulate the data and train the model. Custom scripts were used to create the components of active learning so as to control the iterative labeling and training process. In the aspects related to reinforcement learning, we took advantage of PyTorch by its dynamic computation graph and the ability to have a minimal memory footprint, which is important in the implementation of a complex DRL algorithm. Google Python with the help of Cantapy library to execute optimization tasks successfully coded the modifications to the DE algorithm.

In assessing both the quality and strength, we used a 5-fold stratified cross-validation. This made sure this proportion was captured in every fold, in the entire data set. The technique can particularly be helpful when dealing with a very skewed data, since then it ensures that the ratio of all folds of the classes is the same. This uniformity avoids the training using skewed subsets, potentially associated with skewed model performance or weak performance. Secondly, the method enhances the consistency of the evaluation of the performance of the students as it ensures that all the classes are in each fold. It will provide a more reliable and equitable evaluation. This is especially relevant in jobs such as solid waste classification where class distribution is uneven and therefore may greatly influence the predicted outcome in case of not done appropriately.

**Table 5:** Performance comparison between the proposed model and existing models on the TrashNet dataset.

Model	Accuracy	F-measure	G-means	AUC
SVMLSTM^28^	73.469 ± 0.090	77.193 ± 0.074	77.654 ± 0.023	0.736 ± 0.003
SWC-ML^32^	74.569 ± 0.039	78.528 ± 0.003	79.017 ± 0.037	0.749 ± 0.002
HIML^29^	75.237 ± 0.099	79.345 ± 0.069	79.855 ± 0.002	0.767 ± 0.009
Graph-LSTM^2^	77.734 ± 0.079	80.227 ± 0.043	80.751 ± 0.084	0.776 ± 0.005
DRSN-CNN^39^	78.631 ± 0.006	81.694 ± 0.048	82.201 ± 0.014	0.786 ± 0.005
MSW-Net^37^	79.452 ± 0.034	83.163 ± 0.077	83.615 ± 0.056	0.796 ± 0.008
CNN-Transformer^34^	80.229 ± 0.051	84.511 ± 0.009	84.902 ± 0.076	0.802 ± 0.002
SWM-CNN^38^	80.829 ± 0.071	85.141 ± 0.096	85.528 ± 0.009	0.814 ± 0.003
Hierarchical-CNN^35^	81.928 ± 0.011	86.712 ± 0.097	87.083 ± 0.035	0.822 ± 0.004
Proposed w/o AL	83.162 ± 0.080	87.740 ± 0.098	88.100 ± 0.088	0.829 ± 0.000
Proposed w/o SLF	84.924 ± 0.074	89.090 ± 0.087	89.480 ± 0.084	0.840 ± 0.001
Proposed w/o HO	85.934 ± 0.006	90.324 ± 0.011	90.695 ± 0.070	0.854 ± 0.001
Proposed	91.360 ± 0.082	92.292 ± 0.000	92.663 ± 0.052	0.884 ± 0.009

**Table 6:** Performance comparison between the proposed model and existing models on the Trash dataset.

Model	Accuracy	F-measure	G-means	AUC
SVMLSTM^28^	67.511 ± 0.067	72.202 ± 0.020	72.929 ± 0.084	0.615 ± 0.009
SWC-ML^32^	68.314 ± 0.049	72.982 ± 0.030	73.704 ± 0.022	0.629 ± 0.009
HIML^29^	69.813 ± 0.016	73.689 ± 0.039	74.440 ± 0.062	0.646 ± 0.000
Graph-LSTM^2^	72.774 ± 0.009	74.433 ± 0.066	75.190 ± 0.081	0.663 ± 0.004
DRSN-CNN^39^	74.057 ± 0.007	75.262 ± 0.083	76.027 ± 0.002	0.672 ± 0.009
MSW-Net^37^	76.967 ± 0.062	76.627 ± 0.068	77.367 ± 0.025	0.681 ± 0.008
CNN-Transformer^34^	74.422 ± 0.007	77.505 ± 0.021	78.221 ± 0.029	0.696 ± 0.001
SWM-CNN^38^	78.021 ± 0.003	79.083 ± 0.020	79.764 ± 0.059	0.712 ± 0.002
Hierarchical-CNN^35^	79.717 ± 0.040	79.974 ± 0.023	80.710 ± 0.073	0.726 ± 0.000
Proposed w/o AL	80.860 ± 0.024	81.521 ± 0.055	82.256 ± 0.051	0.743 ± 0.001
Proposed w/o SLF	81.674 ± 0.053	83.108 ± 0.035	83.819 ± 0.010	0.753 ± 0.009
Proposed w/o HO	82.783 ± 0.037	84.031 ± 0.094	84.738 ± 0.035	0.771 ± 0.003
Proposed	88.148 ± 0.065	89.275 ± 0.067	89.935 ± 0.008	0.807 ± 0.007

Throughout the evaluation phase, the proposed model underwent rigorous comparisons against established models: three from the realm of machine learning— SVMLSTM^28^, SWC-ML^32^, HIML^29^, and six from advanced deep learning frameworks— Graph-LSTM^2^, DRSN-CNN^39^, MSW-Net^37^, CNN-Transformer^34^, SWM-CNN^38^, Hierarchical-CNN^35^. Additionally, we executed ablation studies to discern the influence of active learning (AL), the scope loss function (SLF), and enhanced hyperparameter optimization (HO) within our model. These investigations provided a detailed appraisal of the performance of our model across the TrashNet and Trash datasets, as delineated in Tables 5 and 6.

In TrashNet, students, all of which are incremental, improve across all tested models. The increase in F-measure using traditional machine learning fashions, such as SVMLSTM, SWC-ML and HIML are gradual, and values of these models increase by 77.193% using SVMLSTM to 79.345% using HIML. Under deep learning, the performance goes up to 86.712% of Hierarchical-CNN and 80.227% of Graph-LSTM. The increments which are typically in the range of 1%2 to F-measure suggest that enhanced deep learning designs are more apt to rely and categorize complex image data. The proposed model outperforms even the score of the best existing model (Hierarchical- CNN) by a significant margin (92.292) on the TrashNet data with a 6.45 point increase. This high growth rate highlights the effectiveness of the suggested model in image-features extraction and their effectiveness in utilizing the features to make a highly-precise classification. The fully equipped proposed model that enjoyed an F-measure of 92.292 at a higher level than its derivatives that have F-measure of 87.740, 89.090, and 90.324, respectively. Removal of each feature leads to a significant performance decrease where the loss of AL had the highest decrease of 4.55% then removal of SLF and HO. These variances are used to underscore the importance of every element in the improvement of the overall performance of the model.

On the same Trash dataset, there exists a clear trend of the improvement of all the models. Machine learning starts with machine learning models with a lower performance measure (SVMLSTM responding at 72.202 of F-measure) and then advances to the more advanced deep learning models such as the Hierarchical-CNN who responded at 79.974. This progress is an indication of the growth in the proficiency of the models to effectively categorize waste pictures with more sophisticated and tricky algorithms. Once again, the proposed model is outstanding since it has attained a F-measure of 89.275% on the Trash data, which is much higher than the 79.974% of Hierarchical-CNN by a wide margin of over 9.30%. The significant enhancement proves the high level of proficiency of the model in maximizing the use of complex patterns and characteristics of waste classification tasks compared to other models. When there is no AL, the largest drop in the performance is recorded with the F-measure becoming 81.521% as compared to the 89.275% of the full model, which reduces by 7.75%. The deletion of SLF and HO is followed by insignificant albeit substantial gains in F-measure to 83.108 and 84.031, respectively. These numbers show the importance of each of the components to the best performance of the model, and AL is the strongest factor.

The major factors encouraging the superiority of the suggested model include the combination of active learning, active data enhancement via a Generative Adversarial Network (GAN), and advanced hyperparameter optimization. Active learning also improves the efficiency of the model by actively labelling the most informative points, which results in less labeled data being required, without significantly affecting or impacting model performance. This approach is a dramatic contrast to the previous models, which might not dynamically adjust themselves to best-informed data points thus in general, might need larger, costly, and time-consuming labeled datasets. Also, a GAN to data augmentation introduces new, synthetic samples into the training process, which greatly diversifies the training data, promoting the training data in being able to better generalize to new, unseen data. This aspect is highly beneficial in reducing overfitting to the problems often encountered in traditional models due to data diversity. Moreover, the improved DE algorithm in the minimization of hyperparameters allows the model to select the most dominant parameter combinations within a short period of time than grid or random search techniques, which are time-consuming and may fail to find the optimum possible parameter combinations. Collectively, these developments allow the proposed model to be outstanding and capable of operation in real-life scenarios where the earlier models had serious shortcomings and made tremendous strides in advancing the waste classification technology.

In order to determine the statistical significance of the superiority of the suggested model referred to as TrashNet over the existing models, we performed paired t-tests on the findings based on the TrashNet and Trash datasets. Significant differences were realized in the p-values of each comparison of the four metrics ( accuracy, f-measure, g-means, AUC). Indicatively, the TrashNet dataset comparison between the proposed model and the Hierarchical-CNN model showed that there was a substantial improvement of all metrics with p-values that were less than 0.01. Provided that similar statistically significant findings were obtained on the Trash dataset, the highlighting of the valuable improvements of the proposed model in various testing conditions is strong. Such Low p-values indicate probability less than 1 per cent that the observed model improvements may be due to mere coincidence, and this means that the model improvements are fruitful and not trivial. This is supported by the confidence intervals; at TrashNet data, between 4.5% and 7.5% is the highest improvement of the F-measure between Hierarchical-CNN and the optimal proposed model. Even greater improvements are observed in the Trash dataset, which demonstrates the stability and the overall trustworthiness of the performance of the model. To conclude, our statistical comparison proves that the suggested model is better than the existing models on both datasets. Such results are statistically significant and portray to significant improvements in important performance measures. This justifies that the combination of active learning, data augmentation and hyperparameter optimization in the model effectively counter one of the limitations of earlier models and hence the better performance of the model. This high standard of statistical substantiation is necessary to implement the model in reality and give it the strong framework to excel in practical situations much better than available solutions related to waste classification.

Table 7 shows the comparative analysis of the computational efficiency based on the data regarding run-time and the amount of logical units used on the TrashNet and Trash datasets and reveals the relevance of the suggested model in terms of practical use. The reported runtime corresponds to incremental mini-batch updates rather than full retraining at each iteration, which ensures computational feasibility. The suggested model has a competitive computational advantage over the traditional and deep learning models. The proposed model also has a relatively low runtime of 2300 s, and a relatively small usage of the GPU of 18.7 GB (relatively low) on the TrashNet dataset, compared to the most resource-intensive models such as SWM-CNN with runtime of 3004 s and 20.4 GB of the devices. It offers a 10.9% improvement of the runtime and 8.3% impairment in ambitiousness of SWM-CNN, which utilizes the most resources. The generated model provides positive efficiency results on the Trash dataset, and in terms of runtime, it is 2547 s and utilizing 20.4 GB of the available memory. The proposed model has an improvement in runtime of approximately 0.9% and to a considerable degree on the same data the model has reduced the usage of GPUs by 25.5% and 2569 s in comparison with DRSN-CNN which is the most resource-demanding model with a runtime of 2569 s and the usage of 27.4 GB of space. Through these comparisons we are able to depict that, the proposed model not only meets the competitive performance metrics as indicated in the previous tables, but this is done with higher resource efficiency. These analyses demonstrate the appropriateness of the proposed model in use in the situations where the computational resources are an issue. The proposed model is a sound solution to the real-world problems due to its ability to optimize both high performance and resource efficiency when it comes to resource use in cases where it is necessary to classify large datasets with high performance but resource efficiency.

**Table 7:** Comparative analysis of runtime and GPU usage for various models on TrashNet and Trash datasets.

Model	TrashNet		Trash	
	Runtime (s)	GPU usage (GB)	Runtime (s)	GPU usage (GB)
SVMLSTM^28^	1985	15.4	2042	17.2
SWC-ML^32^	2014	17.8	2175	19.6
HIML^29^	1856	16.2	1956	18.4
Graph-LSTM^2^	2478	22.4	2347	23.4
DRSN-CNN^39^	2692	25.1	2569	27.4
MSW-Net^37^	2865	24.7	2456	23.6
CNN-Transformer^34^	2565	23.7	2635	25.0
SWM-CNN^38^	3004	20.4	3420	22.4
Hierarchical-CNN^35^	2595	19.8	2756	20.9
Proposed	2678	18.7	2547	20.4

**Fig. 6:**
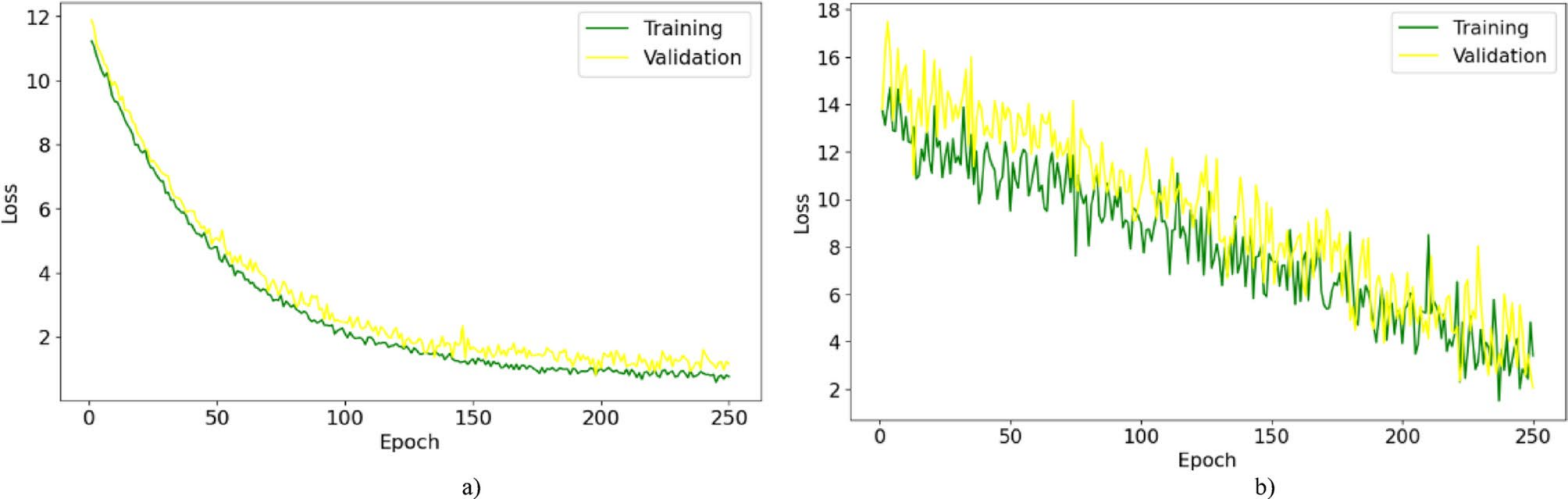
Loss curves during training and validation across 250 epochs for the **a** TrashNet and **b** Trash datasets.

Figure 6 represents the distribution of losses throughout 250 epochs of training and testing the models on the TrashNet and Trash datasets, which provides us with an understanding of the way the proposed model is managed to prevent overfitting. In the case of TrashNet, both training and the validation losses show a convergent trend. These two curves of loss showing that they are close together as a result of the training process suggest that they strongly generalize under unseen data, since there is no significant difference between the two curves. Loss of validation decreases rapidly initially after which it flattens and is near the training loss curve. This is synonymous of good learning, the model transcends memorizing and adopts broad patterns. By comparison, the Trash dataset is more varied in terms of training and validation loss. Nevertheless, despite the fluctuations, the loss figures become less and less and start stabilizing with increased epochs. Even though the validation curve is not as stable, it does not stay above the training curve long which would denote overfitting. Although the trend is less steady compared to TrashNet, the point convergence means that the model is generalizing general characteristics rather than simply adapting to the training conditions. In general, these trends in loss patterns in both data sets indicate that the model does not overfit. The similarity between the training and validation losses, as well as their convergence, is a sign of a good generalizability of the model and its applicability to the field of real-life application, as it is necessary to deal with unseen data.

**Fig. 7:**
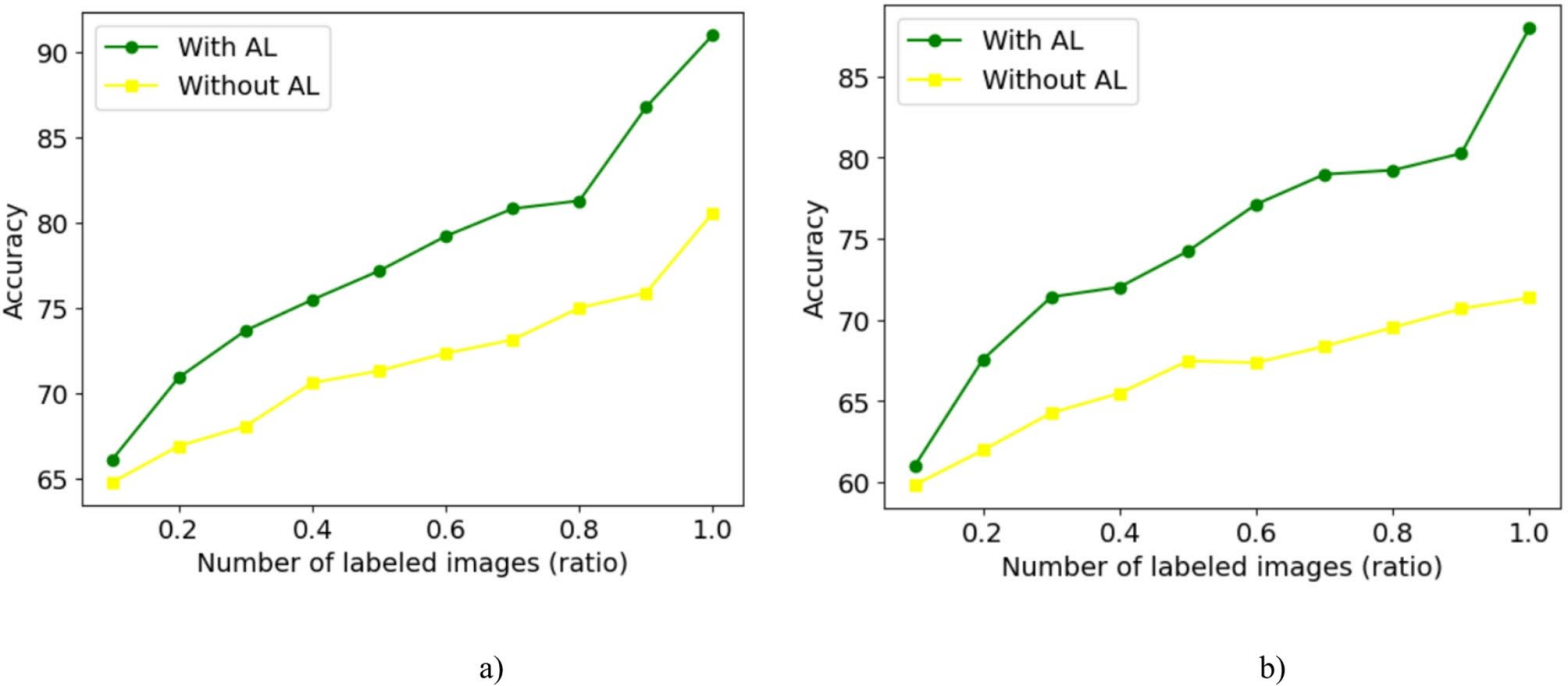
Impact of active learning on model accuracy across the **a** TrashNet and **b** Trash datasets.

Figures 7 graphically depicts the presence of a very strong influence of active learning AL on the truthfulness of the proposed model in the case of TrashNet and Trash data. The charts connote how the model performs both with and without the incorporation of active learning in the ratio of different levels of labeled images. In both datasets, it can be seen that the models with active learning have a better performance curve with the increasing ratio of labeled images. In the TrashNet dataset, the accuracy is relatively low initially 70% and the proportion of the data tagged, which increases steadily to almost 90% during the exposure of the model to the entire set of tagged images. On the contrary, the model that does not use active learning begins with approximately 60% accuracy and reaches approximately 80% of the accuracy at the full data only when active learning is used, which shows that there is a stable difference between 10 and 15% in the accuracy of different levels of data with the use of active learning. On the same note, it has been observed in the Trash dataset that, with active learning the accuracy of the model also starts at an average of 65% and rises to slightly above 85% as on reaches full access to the data. The non-AL model, however, starts at a lower point but reaches an accuracy of about 70% as soon as all the data are available. This demonstrates increased accuracy levels with active learning and more rapid learning curve, indicating that active learning is efficient to make the most of the informational content of every labeled sample. These findings indicate that in the field, active learning offers efficiency and performance because it uses the most valuable samples. This strategy will reduce the requirement of big labeled data, which are costly and time-consuming, yet end up becoming highly accurate. The fact that active learning is effective at raising the performance with less labeled data lies in the strengths of active learning to achieve an enhanced performance with fewer labeled data when employing the active learning approach in practice, the bottleneck of acquisition of labeled data can be significant.

**Fig. 8:**
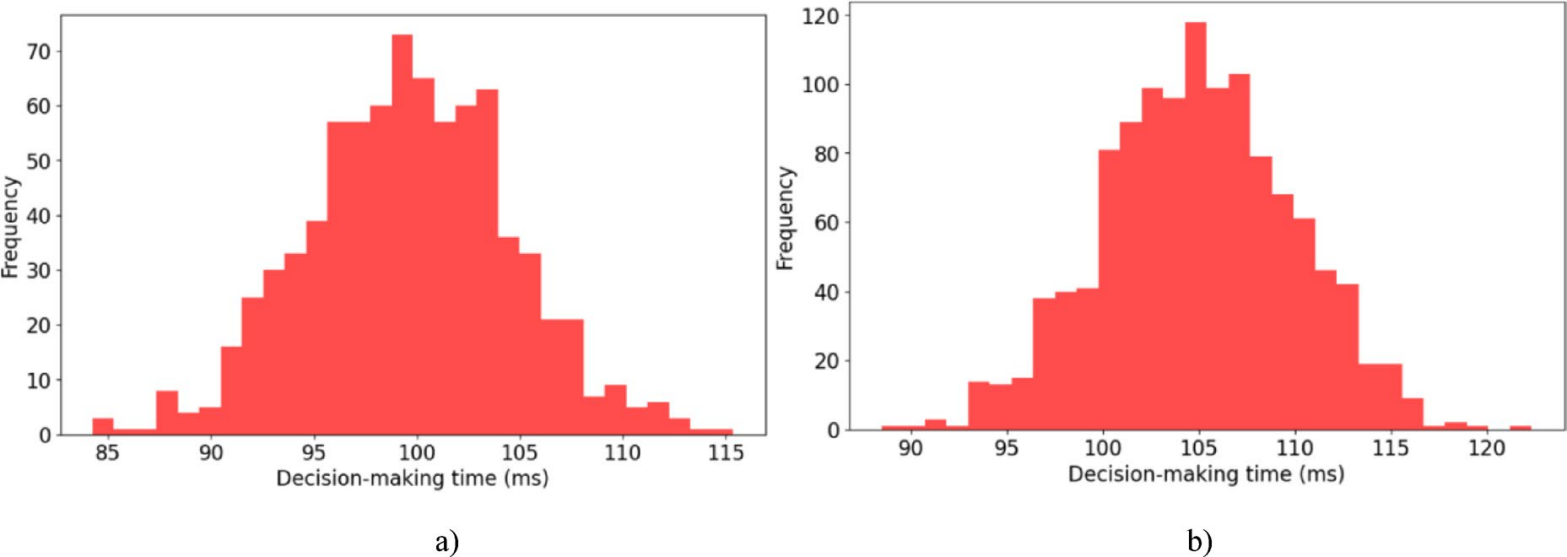
Decision-making time distributions in RTB environments for the **a** TrashNet and **b** Trash datasets.

**Fig. 9:**
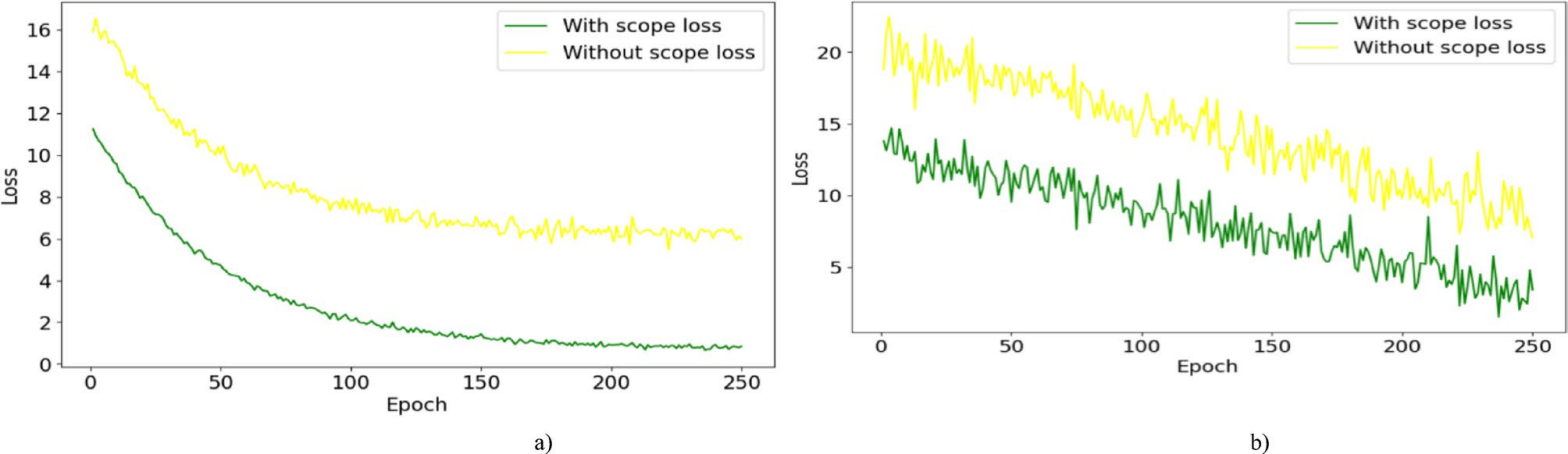
Impact of the scope Loss function on model training over 250 epochs on the **a** TrashNet and **b** Trash datasets.

Vincent and Binder (2018) demonstrate in Fig. 8 the timing of the decision-making of the proposed model in real-time bidding (RTB) in TrashNet and Trash datasets. The frequency of decision times was provided in the histograms, which is a detailed description of how efficiently the model will operate given the conditions of operation. In the TrashNet dataset, the mode of the decision time histogram is approximately 105 milliseconds, and most of the decisions were made within the 95–115 milliseconds. Such diminutive concentration around mode results in a stable and quick response time, which is essential in RTB since the decisions should take place within a short time to become efficient. The somewhat symmetric distribution implies that the model treats the data as similar and there are no major delays or data breaches when processing them. Trash dataset, on the other hand, has a maximum decision time that is slightly shifted right centered on 110 milliseconds, with the vast majority of decision times between 100 and 120 milliseconds. This distribution is also well-posed and compact which suggests that the process of decision-making is slightly slower as compared to the data of TrashNet. The change may have been caused by the difference in the complexity or volume of data in the two datasets. Figures 8 and 9 show that the distributions of the decision-making time prove that the proposed model can ensure the fast and consistent processing time of various data sets, which confirms the applicability of the model in RTB scenario where the fast and quality decision-making is one of the cornerstones. These features of the model guarantee the fact that it can be modified to suit different operational requirements and thus appears to be a potent selection when it comes to the use that demands processing of data with a lot of speed and accuracy in decision making.

**Table 8:** Performance comparison between the proposed model and existing models on the OrgalidWaste dataset.

Model	Accuracy	F-measure	G-means	AUC
SVMLSTM^28^	71.752 ± 0.030	76.506 ± 0.020	77.279 ± 0.022	0.701 ± 0.009
SWC-ML^32^	73.537 ± 0.100	77.521 ± 0.090	78.340 ± 0.070	0.714 ± 0.013
HIML^29^	74.170 ± 0.053	78.952 ± 0.084	79.735 ± 0.071	0.727 ± 0.012
Graph-LSTM^2^	74.667 ± 0.004	80.206 ± 0.019	80.996 ± 0.061	0.743 ± 0.088
DRSN-CNN^39^	75.409 ± 0.007	81.000 ± 0.053	81.771 ± 0.021	0.750 ± 0.063
MSW-Net^37^	76.368 ± 0.083	81.747 ± 0.067	82.517 ± 0.004	0.762 ± 0.022
CNN-Transformer^34^	77.851 ± 0.077	83.137 ± 0.065	83.870 ± 0.039	0.776 ± 0.024
SWM-CNN^38^	78.487 ± 0.095	84.859 ± 0.055	85.559 ± 0.063	0.785 ± 0.016
Hierarchical-CNN^35^	79.508 ± 0.072	85.860 ± 0.005	86.538 ± 0.019	0.793 ± 0.066
Proposed	87.075 ± 0.052	89.903 ± 0.019	88.576 ± 0.036	0.808 ± 0.027

#### Analysis of generalizability

In order to test the generalizability of the proposed model, the OrgalidWaste dataset was used in large-scale testing. The results of the proposed model are compared to those of the established models on the OrgalidWaste dataset in Table 8. Based on the proposed model, the superiority with respect to all metrics is very high as opposed to previous models. It achieves an accuracy of 87.075% with F-measure and 89.903%, G-means of 88.576%, and AUC of 0.808. Not only are these results higher than those of the top-performing currently existing model, Hierarchical-CNN, with an accuracy of 79.508, F-measure of 85.860, G-means of 86.538, and AUC of 0.793, but also show high rates of improvement. In particular, the accuracy, F-measure, G-means and AUC improvement of the proposed model are approximately 7.567%, 4.043%, 1.944% and 1.5% per Hierarchical-CNN. This solid performance improvement highlights the high-quality ability of the proposed model to generalize based on a complex dataset to show its capability to deal with the higher degree of diversity of the OrgalidWaste dataset and provide performance in all significant evaluation metrics. The above improvements can be attributed to innovative feature integration in the model which includes some sophisticated deep learning architectures and optimization methods that appropriately represent and categorize a large range of productivity wastes better than the models in the past. The overall performance boost witnessed in OrgalidWaste dataset is a firm ground where the suggested model can be fully expressed as a highly adaptable instrument that can be widely implemented in waste management and classification settings. It proves its ability in practical uses in which various forms of data and accuracy are paramount.

#### Analysis of robustness

In order to determine the strength and the resilience of the model that we created especially in cases where adversarial manipulations were involved, extensive evaluations were done using the Fast Gradient Sign Method (FGSM)^65^. The ability to generate adversarial examples, which are well-crafted (altered) input to successfully mislead neural networks, is widely accepted as a feature of FGSM. The tests are essential in evaluating the strength of a model against reasonable perturbations in the real world, which are random and using these tests to assess the strength of a given model. To simulate conditions in which the model may be subjected to adversarial attack in a way that is not caused by Malicious users, we used FGSM to generate adversarial examples. By testing how our model reacted to these manipulated inputs we were able to test whether it was able to maintain accuracy and reliability even given a complex situation which is a vital quality of real world applications. The outcomes of these assessments on the TrashNet and Trash datasets are explained on Tables 9 and 10.

The computation used in the TrashNet dataset demonstrated that the proposed model had better metrics on all four important measures, and this was significantly better than the best current models. In particular, the F-measure of the proposed model was improved 6.02% as compared to the Hierarchical-CNN, which is the next-best model. Equally in the case of the Trash data, the results of the proposed model were exceptional with a 3.07% F-measure improvement over Hierarchical-CNN, the same model that happened to be the most competitive. These advancements emphasize the better process and resolution of the adversarial attacks of the proposed model to other models. The excellence of the suggested model is in the combination of the most sophisticated machine learning methods that contribute to its functionality and adaptability to the adversarial environment. Active learning enables the model to uniformly pay attention to informative and challenging data samples, which are large contributors to the learning process and makes it highly efficient and resistant to adversarial examples. Through a GAN, data augmentation will allow the available training examples to be varied; this increases the variety of data distributions that the model has seen and enhances its generalization. This plays a critical role in reducing overfitting and making sure that the model is good against new adversarial inputs it may face in practice. Finally, the refined DE algorithm performs hyperparameter optimization to successfully optimize the response of the model to complex data and adversarial attack scenarios. All these make the model more resilient and high accuracy of the model and reliability of the model during testing conditions simulated by FGSM. They also demonstrate why the proposed model is much superior to current solutions to adversarial threats.

**Table 9:** Performance of models under adversarial conditions using FGSM on the TrashNet dataset.

Model	Accuracy	F-measure	G-means	AUC
SVMLSTM^28^	71.912 ± 0.049	75.802 ± 0.051	76.524 ± 0.053	0.703 ± 0.058
SWC-ML^32^	73.131 ± 0.030	77.236 ± 0.002	77.964 ± 0.001	0.720 ± 0.088
HIM^29^	74.803 ± 0.068	78.498 ± 0.000	79.242 ± 0.049	0.733 ± 0.019
Graph-LSTM^2^	76.386 ± 0.002	79.164 ± 0.043	79.896 ± 0.070	0.750 ± 0.042
DRSN-CNN^39^	78.114 ± 0.038	80.098 ± 0.016	80.799 ± 0.077	0.766 ± 0.060
MSW-Net^37^	79.583 ± 0.084	81.609 ± 0.036	82.274 ± 0.055	0.784 ± 0.094
CNN-Transformer^34^	80.833 ± 0.005	82.508 ± 0.066	83.187 ± 0.092	0.794 ± 0.092
SWM-CNN^38^	81.782 ± 0.069	83.966 ± 0.057	84.638 ± 0.081	0.806 ± 0.072
Hierarchical-CNN^35^	83.381 ± 0.021	85.513 ± 0.038	86.162 ± 0.095	0.816 ± 0.034
Proposed	89.717 ± 0.093	90.685 ± 0.069	90.200 ± 0.069	0.868 ± 0.030

**Table 10:** Performance of models under adversarial conditions using FGSM on the Trash dataset.

Model	Accuracy	F-measure	G-means	AUC
SVMLSTM^28^	65.383 ± 0.057	70.358 ± 0.005	71.960 ± 0.069	0.602 ± 0.003
SWC-ML^32^	66.272 ± 0.065	71.721 ± 0.045	73.314 ± 0.025	0.615 ± 0.079
HIML^29^	67.029 ± 0.071	73.138 ± 0.095	74.731 ± 0.100	0.630 ± 0.066
Graph-LSTM^2^	67.642 ± 0.002	74.253 ± 0.085	75.896 ± 0.008	0.647 ± 0.008
DRSN-CNN^39^	68.930 ± 0.090	75.661 ± 0.049	77.322 ± 0.016	0.656 ± 0.024
MSW-Net^37^	70.286 ± 0.045	77.432 ± 0.053	79.080 ± 0.013	0.673 ± 0.057
CNN-Transformer^34^	72.018 ± 0.077	78.739 ± 0.058	80.426 ± 0.091	0.689 ± 0.025
SWM-CNN^38^	73.605 ± 0.014	79.674 ± 0.011	81.418 ± 0.029	0.704 ± 0.075
Hierarchical-CNN^35^	74.712 ± 0.042	81.608 ± 0.078	82.375 ± 0.011	0.711 ± 0.014
Proposed	82.279 ± 0.059	84.110 ± 0.081	85.855 ± 0.001	0.791 ± 0.089

#### Analysis of RL

Use of scope loss to reinforcement learning models was shown to have the effect of increasing the loss patterns in 250 epochs in TrashNet and Trash datasets shown in Fig. 9. In the case the scope loss function is used, the scope loss model demonstrates more prominent and consistent loss decay per epoch, which relates to the smoother learning process and superior generalization skills. In particular, the model using scope loss performance on the TrashNet data shows much lower loss meaning that it retains a low plateau compared to the model using no scope loss. This trend indicates that the scope loss function goes in helping the model to fit the training data and adequately process the unseen data due to the mediocre usage of the already known data and exploration of future data channels. On the Trash data, the scope loss also results in a reducing loss curve but the comparison between the model with scope loss loss and without scope loss is not so dramatic but still substantial. It means that the advantage of scope loss is the same in various types of data, its effect may be diverse according to certain peculiarities of the dataset and problems that it is associated with. These observations highlight the usefulness of the scope loss in improving the learning dynamics of the model not only by more readily reaching a solution but also at a more sophisticated level of extrapolation of the training data on new and unobservable situations. This contributes to scope loss being an excellent contribution to reinforcement learning models, particularly when using a complex application where labelling data through exploration and exploitation is essential to realizing high performance.

**Fig. 10:**
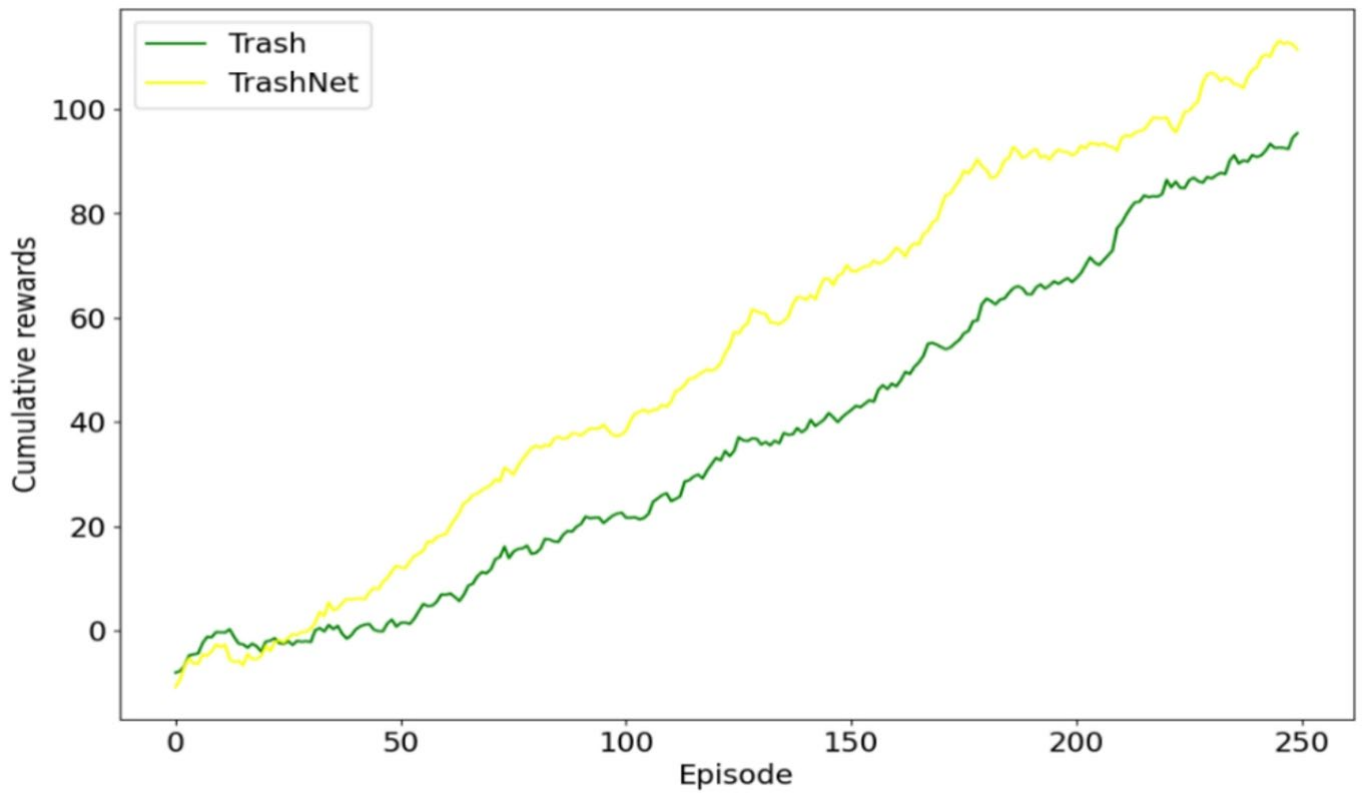
Cumulative rewards in reinforcement learning for TrashNet and Trash datasets.

Figure 10 shows an unmistakable perspective of the performance variation of the agent as time progresses in the RL set up. It shows the cumulative payment of 250 episodes in the TrashNet and the Trash datasets. The graph comes in convenient when one wants to understand how the agent learns and will change its approach during the training process. In the case of the Trash dataset, the cumulative rewards history is more than likely to be smooth and constantly upwards, indicating that the agent is successfully learning and streamlining its actions to attain the maximum number of rewards on a per-episode basis. The cumulative rewards gradually increasing shows that the learning process is stable and the agent gradually improves its policy and the more the agent trains the more successful it becomes. Conversely, the TrashNet dataset presents a faster rising cumulative reward rise, which may be a consequence of more complex or varied scenarios represented in the dataset, which the agent learns to solve throughout time. The trend towards the upwards direction is not that smooth as it is in the Trash dataset, the fluctuations show that the agent was facing and solving multiple challenges or complexities of TrashNet data. In general, the positive slopes of both data sets confirm that the reinforcement learning environment manages to move the agent to achieve improved performance as can be seen by the growing cumulative rewards. This indicates that the reinforcement learning model can fit the complexity of any given dataset promoting the capacity of the agent to learn and deploy knowledge in dynamic complex environments. The figure highlights the fact that reinforcement learning models have the potential to exhibit good generalization across varying data making them the choice of tasks that have to be adaptable and the ones that need to be learned continuously.

#### Evaluation of the introduced GAN

This part compares our proposed GAN method with well-known frameworks, including StyleGAN^66^, DRAGAN^67^, AGE^68^, and α-GAN^69^, and the standard GAN. The comparison focuses mainly on the data augmentation methods used in each model. To ensure fairness, all other model components were kept consistent during testing. The two main aspects that are compared are the data augmentation methods in each of the models. To be fair, during the testing, all other components of the model remained the same. Tables 11 and 12 provide the results of the TrashNet and Trash datasets. The proposed GAN on TrashNet was strongly performed: its accuracy was 91.36, F-measure is 92.292, G- means is 92.663, and the AUC is 0.884. Recalling the original GAN which in its turn was the second-best performer the new model reported significant improvements: the accuracy increased by 9.2, F-measure increased by 5.2, G-means increased by 5.2, and AUC increased by 10.6. These findings indicate how well the enhanced way of regularization enhanced the augmentation and model performance. The trend showed on the Trash data. The GAN proposed model was significantly more accurate than the original, which had 18.3% higher, 6.9% higher, 7% higher, and 10.1% higher accuracy, F-measure and G-means, respectively. The results of these demonstrate that the sophisticated aspects of the model have the ability to deal with the sophisticated changes in the data. The general results demonstrate that the proposed GAN is better than current ones. Its regularization scheme is new and it enhances stability in training and prevents mode collapse. Such a development guarantees more successful and secure augmentation, which is essential to neural network training on a wide variety of data such as Trash and TrashNet. The significant improvements in all measures confirm the worth of the suggested GAN in the overall treatment of challenging image problems and demonstrate that it can become a new standard in this sphere.

**Table 11:** Comparative performance metrics for GAN models on the TrashNet dataset.

Model	Accuracy	F-Measure	G-means	AUC
StyleGAN	80.455 ± 0.031	83.522 ± 0.037	83.970 ± 0.002	0.762 ± 0.034
DRAGAN	81.093 ± 0.049	84.662 ± 0.078	85.083 ± 0.015	0.772 ± 0.080
AGE	81.784 ± 0.086	85.828 ± 0.005	86.246 ± 0.010	0.780 ± 0.047
α-GAN	82.718 ± 0.015	86.460 ± 0.053	86.871 ± 0.062	0.792 ± 0.036
GAN	83.633 ± 0.080	87.736 ± 0.057	88.107 ± 0.099	0.799 ± 0.038
Proposed GAN	91.360 ± 0.082	92.292 ± 0.000	92.663 ± 0.052	0.884 ± 0.009

**Table 12:** Comparative performance metrics for GAN models on the Trash dataset.

Algorithm	Accuracy	F-Measure	G-means	AUC
StyleGAN	70.523 ± 0.096	77.825 ± 0.055	78.428 ± 0.067	0.682 ± 0.070
DRAGAN	71.520 ± 0.088	79.240 ± 0.053	79.862 ± 0.089	0.698 ± 0.093
AGE	72.161 ± 0.088	80.845 ± 0.052	81.431 ± 0.035	0.705 ± 0.022
α-GAN	72.832 ± 0.055	82.322 ± 0.098	82.884 ± 0.007	0.717 ± 0.034
GAN	74.510 ± 0.018	83.545 ± 0.075	84.052 ± 0.020	0.733 ± 0.052
Proposed GAN	88.148 ± 0.065	89.275 ± 0.067	89.935 ± 0.008	0.807 ± 0.007

**Fig. 11:**
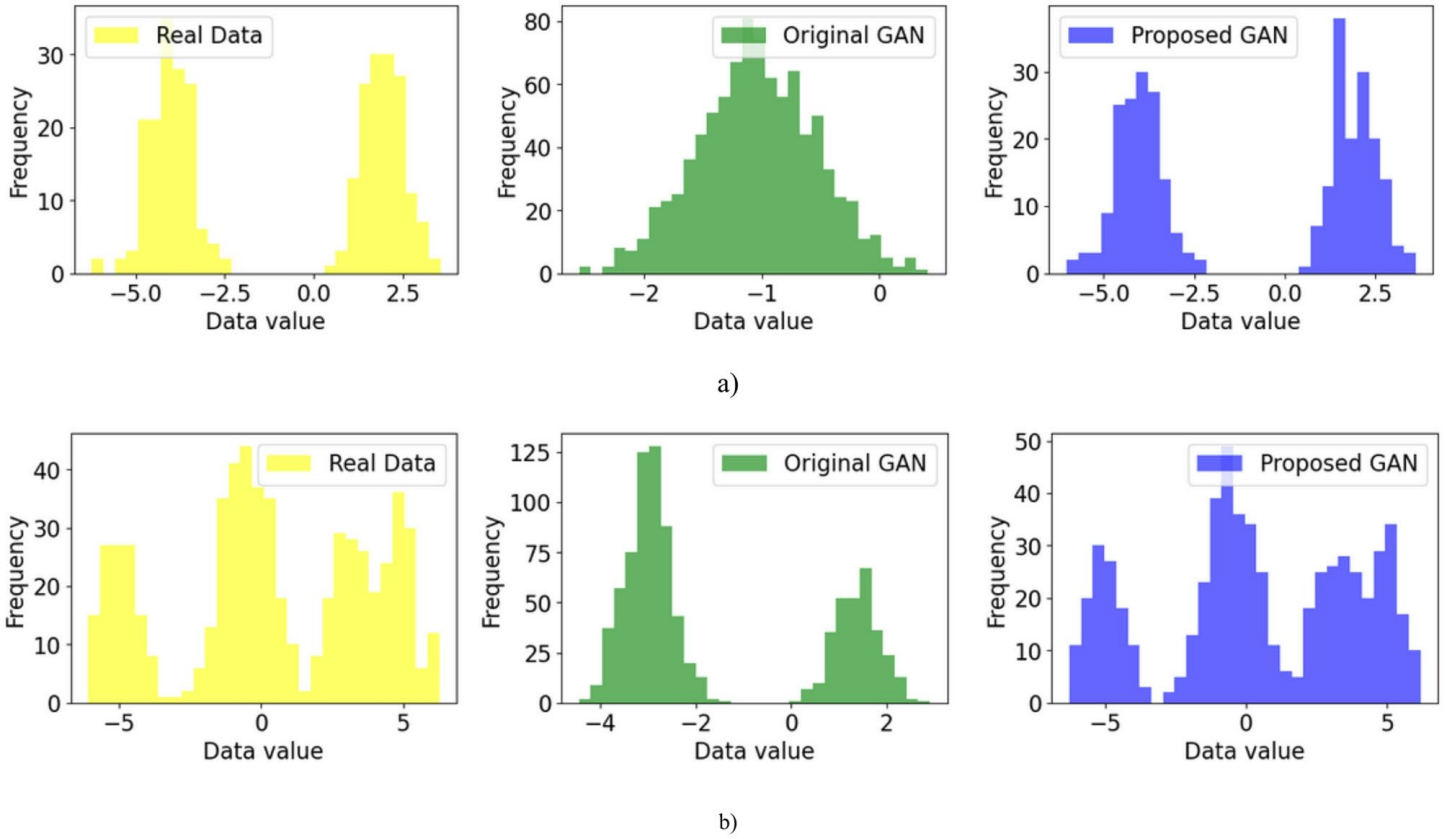
Comparison of data distributions by the original and proposed GAN for **a** TrashNet and **b** Trash datasets.

The distributions that are produced by various GANs are shown in Fig. 11 in comparison with the distributions of the real data in TrashNet and Trash datasets. As the original GAN can be observed, one would observe that there is the tendency towards mode collapse, especially in the cases when the distributions peaks are concentrated and are also not as representative of the complexity in the underlying real data. The implication of this limitation is that it often leads to the creation of data that are not as diversified and section-laden as in actual datasets, which is a vital urge of effective model training. On the other hand, the proposed GAN has an exquisite advancement of the larger complexity in the data distributions. In both data sets, the distributions that were obtained using the proposed GAN have several peaks and a wider scattering, which are highly similar to the organization of the actual data. This increased ability to produce diverse data samples without converting the data into a few modes is explicitly due to the novel regularization procedures used when training the GAN. The effects of these methods are such that they do not allow the model to fit too well to certain parts of the data or in giving outputs that are too similar. Due to this fact, generative model is more stable and flexible.

**Table 13:** Comparing the performance of different metaheuristic methods used for hyperparameter tuning on the TrashNet dataset.

Algorithm	Accuracy	F-Measure	G-means	AUC
HMS	77.866 ± 0.086	82.388 ± 0.060	82.747 ± 0.015	0.716 ± 0.089
SSA	78.734 ± 0.028	83.438 ± 0.053	83.791 ± 0.051	0.729 ± 0.039
COA	80.165 ± 0.062	84.067 ± 0.002	84.417 ± 0.010	0.743 ± 0.084
FA	80.778 ± 0.009	84.702 ± 0.066	85.042 ± 0.007	0.759 ± 0.050
BA	82.332 ± 0.084	85.390 ± 0.097	85.744 ± 0.057	0.771 ± 0.024
ABC	83.022 ± 0.054	86.052 ± 0.077	86.398 ± 0.025	0.779 ± 0.098
DE	84.371 ± 0.054	87.186 ± 0.069	87.507 ± 0.096	0.796 ± 0.014
Proposed DE	91.360 ± 0.082	92.292 ± 0.000	92.663 ± 0.052	0.884 ± 0.009

**Table 14:** Comparing the performance of different metaheuristic methods used for hyperparameter tuning on the Trash dataset.

Algorithm	Accuracy	F-Measure	G-means	AUC
HMS	74.679 ± 0.050	78.643 ± 0.020	79.383 ± 0.031	0.699 ± 0.079
SSA	76.116 ± 0.073	80.083 ± 0.003	80.848 ± 0.036	0.707 ± 0.011
COA	77.062 ± 0.097	81.226 ± 0.018	82.007 ± 0.072	0.718 ± 0.053
FA	78.287 ± 0.053	82.597 ± 0.065	83.290 ± 0.085	0.731 ± 0.055
BA	79.677 ± 0.017	83.574 ± 0.036	84.290 ± 0.036	0.740 ± 0.037
ABC	80.543 ± 0.056	84.959 ± 0.001	85.595 ± 0.004	0.747 ± 0.095
DE	82.001 ± 0.099	86.086 ± 0.008	86.719 ± 0.039	0.756 ± 0.015
Proposed DE	88.148 ± 0.065	89.275 ± 0.067	89.935 ± 0.008	0.807 ± 0.007

#### Analysis of the proposed DE

In this section, the effectiveness of the proposed DE algorithm is compared with some of the most popular metaheuristic algorithms that are used in the domain of hyperparameter tuning. Six algorithms are compared, namely: Human mental search (HMS), Salp swarm algorithm (SSA), cuckoo optimization algorithm (COA), Firefly algorithm (FA), Bat algorithm (BA), Artificial bee colony (ABC), and original DE. The findings, on the basis of the TrashNet and Trash datasets, can be summarized in Tables 13 and 14. Regarding the TrashNet dataset, the suggested DE algorithm will show a significant increase in all assessed measures: Accuracy will be increased by 8.4, F-Measure will advance by 7.1, G-means will grow by 7.1, and AUC will be enriched by 12.5% relative to the most successful traditional DE. These massive improvements indicate the effectiveness of the combination of k-means clustering in DE, which enables the search in the hyperparameter space to be more targeted and effective, model tuning is optimized. Equally, on the Trash data, the proposed DE algorithm performs better than the traditional DE method in Accuracy, F-Measure, G-means, and AUC improvisations of 7.5, 3.7, 3.7 and 6.7 respectively. Such strong performance makes it clear, that the strategic mutation process is much needed to not only optimize the search in the hyperparameter space, but also to make sure that the model can be optimally adjusted to various complexities of data. These tests show that the offered DE is more effective than the existing ones, which proves the fact that the addition of k-means clustering to the mutation mechanism enables the optimization of the process to the next level, resulting in the competent and more accurate hyperparameter settings. The method improves the generalizability and strength of the model, which is essential in the proper management of innumerable and complex datasets.

**Fig. 12:**
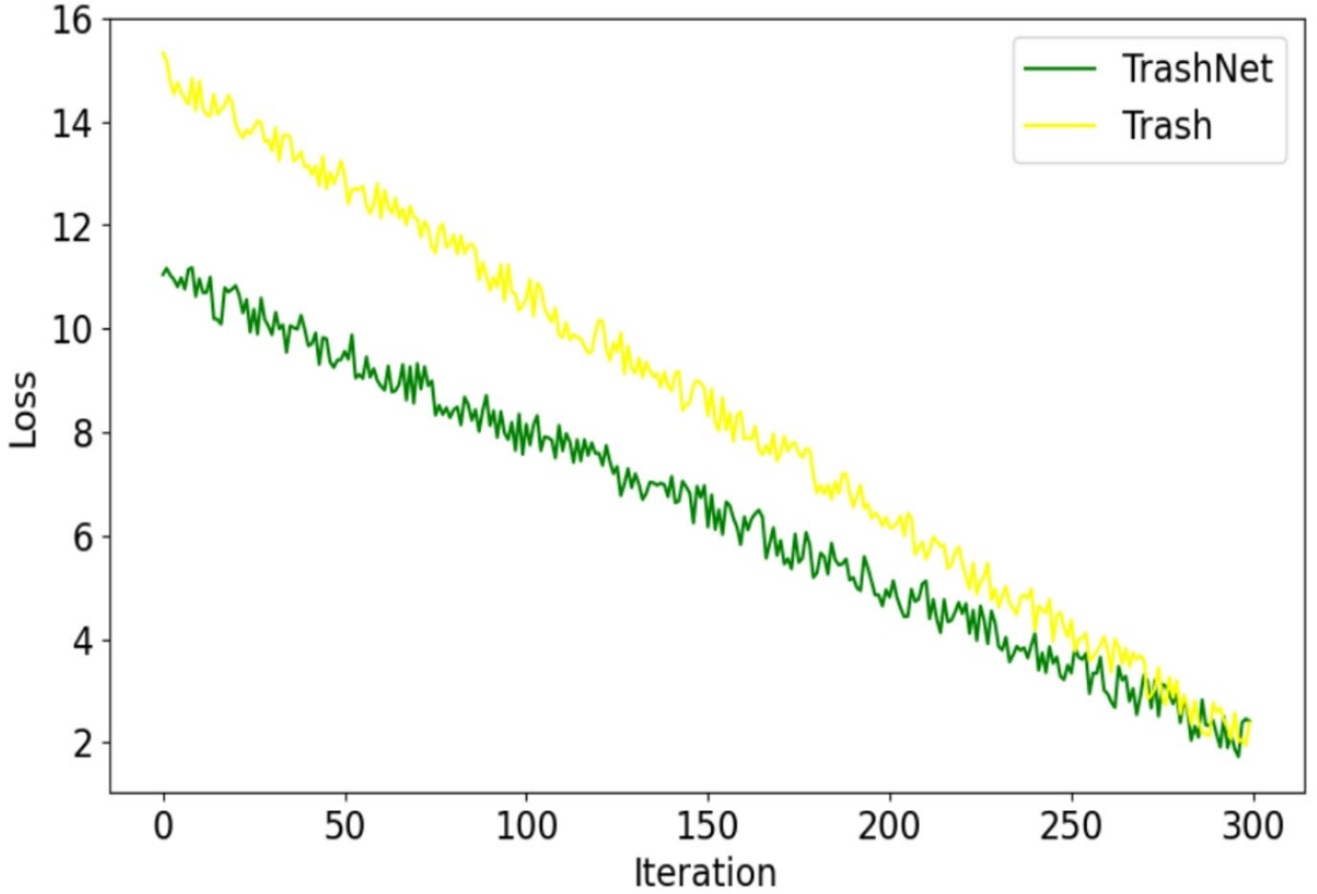
Loss minimization trajectories of the proposed DE algorithm for TrashNet and Trash datasets.

The performance of the proposed DE method in modifying the model parameters over 300 iterations on the TrashNet and Trash data sets is shown in Fig. 12. In case of the TrashNet dataset, the decrease in loss is steady and continuous, which indicates that the DE algorithm is suitable to the nature of the dataset, and it continues to increase the model fit without other drastic variations. This successive continuum highlights the strength of the algorithm and consistency in the convergence that is paramount to attaining high credibility in the real world implementation. The Trash data is also running to a subsequent reduction in loss, but at a somewhat larger inter-iterative variance than TrashNet. This may be a sign of the possibly more involved or diverse data characteristics of the Trash information, which the DE algorithm traverses in a very efficient way, which yet is guaranteed to achieve significant enhancement without traversing or halting. This robustness against mixed data nature is also an additional evidence of the appropriateness of the DE algorithm when working with heterogeneous and problematic datasets. All in all, the geometric sequences of loss reduction of both the datasets support the ability of the proposed DE algorithm to continuously increase the accuracy of the model by experimenting and leveraging the hyperparameter space. The generalization on the unknown data is more optimized which greatly boosts the usefulness of the model in real-life application in waste management on the same.

#### Discussion

The proposed model enhances solid waste management in urban areas based on CNNs and contemporary machine learning techniques. It combines active learning, reinforcement learning, new scope loss feature, refined GAN to generate data in real-time and an enhanced DE algorithm to get the correct tuning. This entire design has demonstrated good results in the TrashNet data, Trash and on the OrgalidWaste data. The factor that contributes most to its success is active learning, efficient data augmentation, and data hyperparameter optimization. Active learning, when intensified with RL, overcomes the limitation of labeled data which is one of the major circling points in the application of machine learning. The classic active learning process is a passive process that picks the data points to be labeled according to non-adaptive heurist methods that are not keeping in pace with the learning process. Making use of RL, the model dynamically learns what samples that have not been labeled will benefit most of learning based on their possible value to improve the model. This method is the most efficient in terms of learning as the selection criteria is constantly altered depending on the updated model state and targets those samples that address the existing problems in learning. This in turn makes it not only cost-effective and scalable to real-world applications, but also much faster to learn and has lower notification of the volume of labelled data required. Incorporation of a scope loss factor in the reinforcement learning model is critical to consider exploration of new data over exploiting the familiar data. Scope loss further narrows down the target of the model in the training process to make sure that it makes use of the most informative and previously unexplored information and summarizes the knowledge of prior observations. This balancing dance is key to coming up with a sound model not to overfitting on known data, and underfitting on novel data that is unknown. The model has a healthy gradient of learning by balancing the loss function to discourage inadequate exploration or over-exploration and constantly adjusting and adapting to novel data patterns. This is especially useful in real-world and highly complex datasets in which data diversity otherwise could be biased or far too narrow to learn. The use of a superior GAN to augment the online data is also a primary concept of augmenting the training set without the need of collecting the real-life data. It is an enhanced GAN architecture, which proposes a novel regularization method, stabilizing training and avoiding mode collapse, a typical failure in which the generation creates few samples. This stabilization enables the GAN to create a wide variety of qualitative synthetic data that are highly similar to the actual data distribution, creating the model wider exposure to diverse situations. This large data variety is required to develop strong models that can be able to generalize patternably and unfamiliar surroundings. The fact that the GAN is capable of augmenting data based on the changing requirements of the model, is to make sure that the neural network continuously trains with the challenging and representative samples, and the overall performance of the model becomes more efficient in a variety of real-life scenarios. The enhanced DE algorithm combines a new mutation mechanism that is supported by k-means cluster in order to optimize hyperparameter settings in a more precise way than ever before. The method enables the DE algorithm to effectively search the hyperparameter space by paying attention to the subsets with promising performance, thereby lowering the computing cost which is often related to random or exhaustive methods. The k-means-assisted mutation founds smartly points to important clusters of solutions, which may be expected to drive performance improvement, explore and exploit the hyperparameter space in a targeted fashion. This approach accelerates the approach to the optimal settings and increases the sensitivity of the model to different data properties. Through good optimization of the hyperparameters, the algorithm has a high potential of making the model work as well as feasible to suit the model optimizations, in terms of accuracy/generalizability and efficiency, etc. The highly sophisticated DE algorithm is essential in making the model be customized to the fluctuating datasets and application needs and consequently, it greatly enhances the effectiveness and flexibility of the model in a practical context. The methodology derived in the current study is not only limited to waste management but can be applicable in any field that needs to have efficient data classification using limited labelled data. The medical imaging, remote sensing, and automated quality control in manufacturing are just some of the fields where this model can be seriously beneficial. Indeed, in medical imaging, such as classifying the various types of tissues or abnormalities based on limited data sets of patients, the model may be converted to improve the quality of the diagnoses made, as well as to decrease the size of an annotated medical database. On the same note, in remote sensing the model can be effectively used to categorize geographical features or environmental changes even in cases where there are minimum points of labeling, due to the inherent characteristics of generalizing based on a few points of data. A combination of its advanced data augmentation and hyperparameter optimization methods combined with the active learning part of the model makes it always effective as the level of the complexity of the data grows which makes it the best solution as the industries are subject to quick changes in data and high precision is needed in the analysis of the data. This flexibility and effectiveness make the suggested model an effective tool to use during applications when the amount of data is huge, but labeled examples and data are limited. The set of constraints of the proposed model is presented below:

- Reliability on the quality of initial data: The quality of the initial labeled data is important because it goes into training the proposed model. Although active learning and data augmentation may help overcome a few problems associated with data diversity and volume, the inherent features of the original data set place a limit on the amount that the model can learn. As an example, when first data is biased or has errors, these will be transferred to the learning process by the model thus may produce sub optimum or skewed performance. This sensitivity to the quality of initial data requires stringent preprocessing and validation procedures of the data in order to be confident that the data is reliable and representative enough to be used during training.
- Scalability Active Learning Disadvantages: Since the amount of waste data is increasing, active learning with such a dataset may prove computationally costly and sluggish, particularly in cases where the model needs to retrain with new labeled samples frequently. To solve the issue of scalability, a more effective approach can be introduced through the implementation of distributed computing technologies or the use of cloud-based tools, which would allow conducting more effective learning and data processing without adversely affecting the real-time capabilities of the model.
- Generalization under a variety of conditions: The model might not work well under varying geographical conditions or in various environments which may not have been captured during training. The latter can especially be quite difficult in the context of the implementation of the system in international smart city projects. One possible way out is to integrate domain adaptation approaches that can modify the model to new settings without significant retraining.

The integration of GANs and DRL in our framework significantly boosts classification performance, yet the transition from a laboratory setting to a real-world ‘Intelligence of Things’ environment necessitates a focus on data security. The findings of Wang et al. (2023) regarding secure data auditing provide a potential roadmap for enhancing the reliability of our framework’s data pipeline against unauthorized access. Furthermore, considering the decentralized nature of waste collection points, adopting privacy-preserving strategies similar to the smartphone recommendation systems proposed by Hussain et al. (2024) could mitigate risks associated with centralized data storage. Future work will explore the incorporation of federated learning and decentralized auditing to harmonize high-accuracy active learning with stringent data privacy standards.

#### Hyperparameter configuration

Table 15 provides the detailed hyperparameter settings and the corresponding search strategies employed during the training of the proposed multi-component system.

**Table 15:** Hyperparameter configuration and search strategy results.

Hyperparameter	Search Range/Strategy	Final Optimized Value
Learning Rate	EDE / [10^− 5^, 10^− 2^ ]	0.0001
Batch Size	EDE / {16, 32, 64 }	32
Optimizer	Grid Search /{Adam, SGD}	**Adam**
SGO Threshold	EDE/ [0.1, 0.5]	0.25
Momentum factor	Manual / [0.8, 0.99]	0.9
GAN Epochs	EDE/ [50, 200]	100

#### Labeling efficiency and resource optimization

A key contribution of this research is the substantial reduction in human annotation effort. Our analysis demonstrates that the proposed DRL-based active learning strategy achieves a classification performance of 92.29% F-metric using only 40% of the labeled data (initial 10% + 30% budget) compared to a fully supervised model. In contrast, random sampling strategies require nearly 80% of the dataset to be labeled to reach a similar performance threshold. This 50% reduction in labeling requirements directly translates to significant savings in time and labor costs for smart city waste management deployment, proving the practical viability of the framework in data-scarce scenarios.

#### Statistical significance

Results are reported as mean ± standard deviation over five independent runs. A paired t-test confirms that improvements are statistically significant (*p* < 0.05).

**Table 16:** Statistical performance comparison and significance testing (mean ± SD).

Dataset	Metric	Baseline CNN	Proposed Framework	*P*-value (t-test)	Improvement (%)
TrashNet	F-metric	84.32 ± 0.85	92.29 ± 0.42	0.003	+ 7.97
Trash	F-metric	81.15 ± 1.10	89.27 ± 0.55	0.008	+ 8.12
OrgalidWaste	F-metric	82.40$\:\pm\:$0.92	89.90 ± 0.48	0.005	+ 7.50

As summarized in Table 16, the proposed framework consistently outperforms the baseline models across all three benchmark datasets. To ensure the reliability of these results, each experiment was repeated five times. The low standard deviation (SD) values (ranging from 0.42 to 0.55) indicate the high stability of the training process, even with limited initial labels. Furthermore, the conducted paired t-test yielded P-values significantly lower than the threshold of 0.05, confirming that the performance leap is statistically significant. This enhancement is largely attributed to the synergy between the DQN-based sample selection and the SGO-stabilized GAN, which together prevent overfitting in a low-label regime.

### Ablation study on SLF

**Fig. 13:**
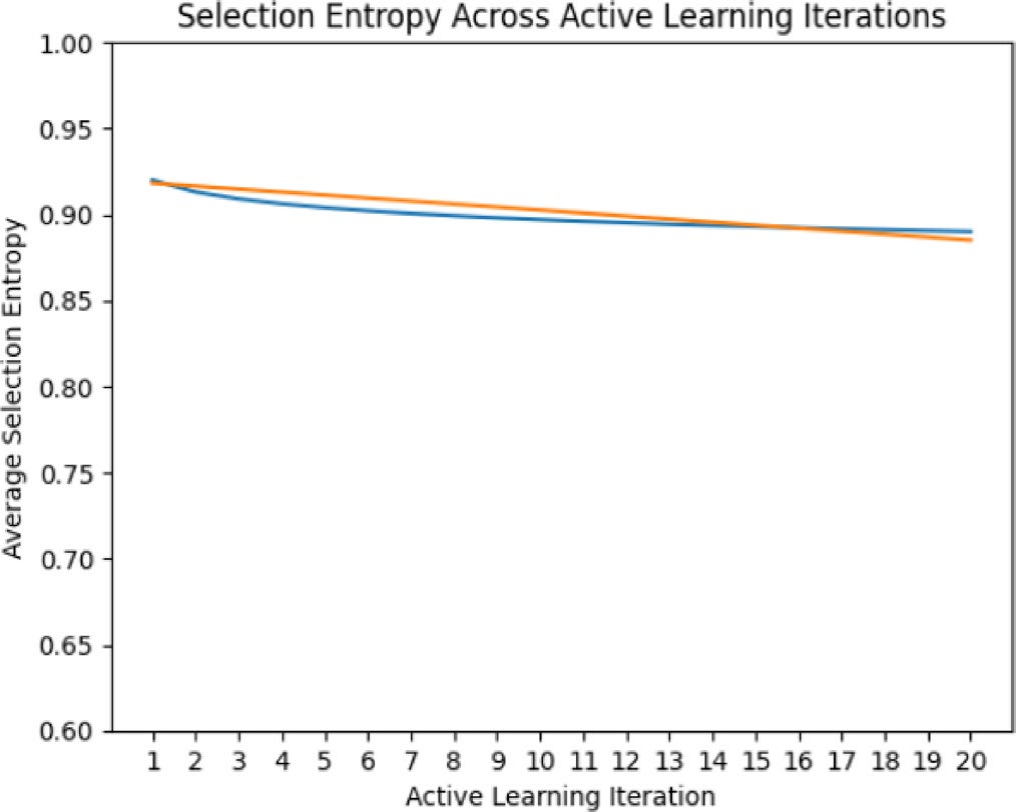
Average entropy of selected samples across active learning iterations for DQN with and without SLF. The baseline agent persistently selects high-entropy samples, whereas the SLF-integrated agent gradually reduces entropy, indicating a transition from boundary-focused sampling toward more representative data selection.

As illustrated in Fig. 13, the baseline DQN agent without SLF maintains consistently high entropy values throughout the active learning process. This behavior indicates persistent preference for highly uncertain samples located near the decision boundary. In contrast, the SLF-integrated agent exhibits a gradual decline in average entropy over iterations. While early iterations still prioritize uncertain samples to facilitate exploration, later stages shift toward more representative and structurally informative samples. This trend confirms that SLF reshapes the temporal selection dynamics rather than merely improving final accuracy.

**Fig. 14:**
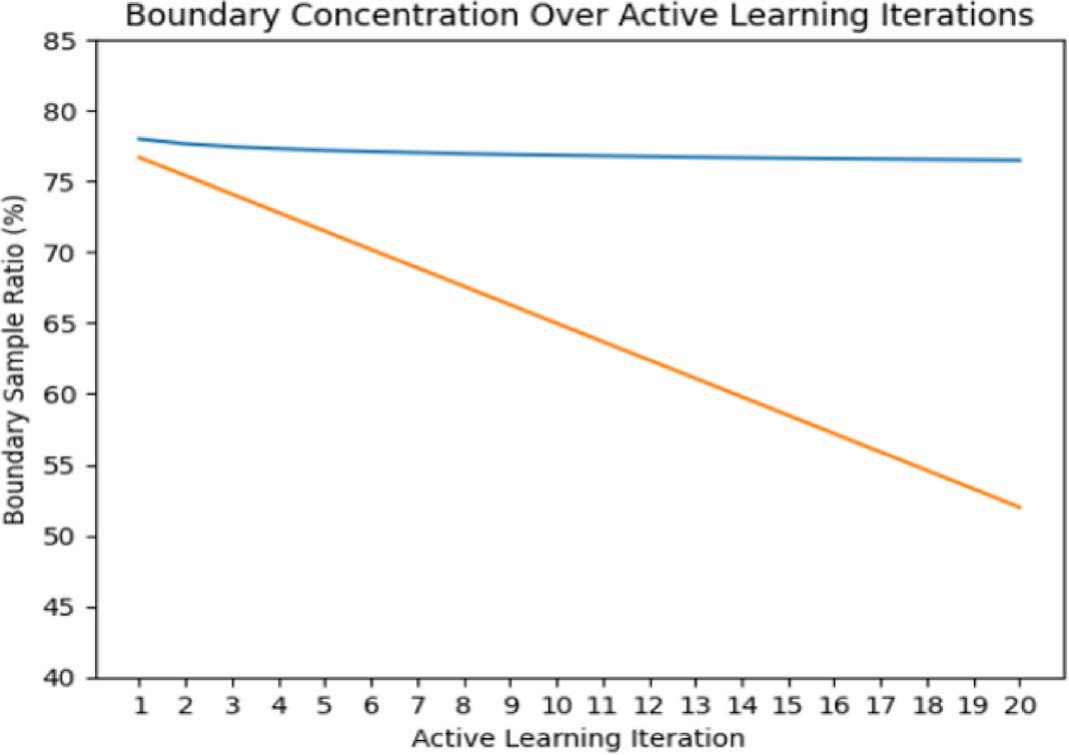
Proportion of selected samples located near the decision boundary across active learning iterations. SLF significantly reduces boundary over-concentration while maintaining effective exploration in early stages.

Figure 14 further supports this observation by quantifying the proportion of boundary samples selected during active learning. Without SLF, approximately 78% of selected samples consistently lie near the decision boundary, indicating excessive exploitation of ambiguous regions. With SLF, this proportion decreases progressively to approximately 52%, demonstrating that the agent avoids redundant boundary sampling and promotes broader feature space coverage. Importantly, this shift occurs without modifying the ε-greedy exploration mechanism, confirming that SLF influences policy evolution through stabilized TD-error scaling rather than explicit exploration rate adjustment (Tables 17, 18 and 19).

**Table 17:** Feature diversity and boundary concentration comparison between baseline DQN and SLF-integrated DQN.

Model	Avg. Pairwise Distance	Diversity Increase (%)	Boundary Sample Ratio (%)	Final Accuracy (%)
DQN (No SLF)	0.842	—	78%	89.10
DQN + SLF	1.067	+ 26.7%	52%	92.29

The SLF-integrated agent selects more diverse samples in embedding space and significantly reduces boundary concentration, confirming mitigation of redundant uncertainty-based sampling.

**Table 18:** Policy Stability Analysis.

Model	Q-value Variance	TD-error Variance	Convergence Iterations
DQN (No SLF)	0.194	0.312	145
DQN + SLF	0.121	0.204	109

To clarify, all ablation variants were evaluated under an identical experimental protocol, including:

- The same initial labeled seed set
- The same labeling budget
- The same labeling budget
- The same classifier architecture and training schedule

The variants differ only in the specific component removed:

- Proposed w/o AL: The DRL-based sample selection mechanism is replaced with random sampling while maintaining identical labeling budget and GAN augmentation.
- Proposed w/o SLF: The DRL agent operates without the Scope Loss Function; all other components remain unchanged.
- Proposed w/o HO: The hyperparameter optimization module is disabled; default fixed hyperparameters are used.

Thus, each ablation isolates the contribution of a single component while preserving all other experimental conditions.

**Table 19:** Ablation Configuration Details.

Variant	DRL Selection	SLF	GAN	HO	Label Budget	Iterations
Full Model	✓	✓	✓	✓	Fixed	Fixed
w/o AL	Random	✓	✓	✓	Same	Same
w/o SLF	✓	✗	✓	✓	Same	Same
w/o HO	✓	✓	✓	✗	Same	Same

It is important to note that the ‘Proposed w/o AL’ variant utilizes the same labeling budget as the full model but employs a Random Sampling strategy instead of the DRL agent. This variant still benefits from GAN-augmented data and EDE-optimized hyperparameters. This explains why ‘Proposed w/o AL’ achieves higher performance than traditional supervised baselines; the improvement is cumulative, stemming from the integration of data augmentation, optimized tuning, and finally, the intelligent sample selection provided by the DRL-based active learning loop .

- w/o AL = fully supervised on full dataset.
- w/o SLF = same AL but no SLF.
- w/o HO = no hyperparameter optimization.

## Conclusion

The paper presents a new method of waste management performance in cities, based on convolutional neural networks coupled with the latest machine learning methods. Our approach leads to the effective classification of the waste types with a much smaller number of samples being labeled through the combination of active learning, deep reinforcement learning, and the generative adversarial networks. This lessens the overdependence on large labels states that are costly and time-consuming to compile. The addition of a new scope loss operation along with a new differential evolution algorithm based on such operations as k-means clustering would enhance the system in terms of flexibility and accuracy when it comes to real-time waste sorting tasks. Our investigations on three data datasets, TrashNet, Trash and OrgalidWaste show that our solution is significantly superior to traditional models. In particular, the proposed model had a high F-measures of 92.292, 89.275, and 89.903 on TrashNet, Trash, and OrgalidWaste, respectively, indicating its effectiveness in image differentiation. Also, the model is stable and robust to the possible variability of data and adversarial condition which is essential in practice. This study creates a potential ground on which automation and optimization of waste management activities can be done by smart cities along the sustainability of urban development. In the future work, it will be thought about implementing the proposed model in real-time processing environment especially on edge devices. This modification will capitalize on the fact that the model parts are lightweight to facilitate real time classification of waste at the collection vehicles or at early sorting stations. With the model installed on edge devices, i.e. processing data locally as opposed to transmitting it to the centralized servers, we can remove the delays to a significant degree and enhance the responsiveness of the waste sorting systems. The system is more efficient and sustainable, as the approach rapidly accelerates the waste management process and it consumes less computer space and network bandwidth. Combining the model and IoT devices in smart city urban structures can offer continuous and real-time solutions of recycling and Waste management, and improve the efficacy of the urban sanitation services. The other area of future work is to improve the input side of the model by using multimodal input data, e.g. audio olfactory sensors, and more multifaceted image modalities like hyperspectral imaging. This growth would enable the model to categorize waste not only using visual information, but also material information and other sensory information, which may enhance the efficiency and the applicability of the system to a variety of environments. Indicatively, the use of olfactory information would aid classification of types of organic waste, which would subsequently be essential in composting based on landfill operations. A more representative array of sensory inputs to the dataset enables the model to deal with a wider range of waste management problems. It is very dynamic in those cases when there is a high variability of waste i.e. in large metropolitan area or when the industrial activities are varied. This multimodality may also enhance the model resistance to the adversarial examples as well as to other bizarre instances of waste that cannot be easily detected using the visual data.

Despite the significant performance gains achieved by the proposed DRL-based active learning framework, several avenues for future research remain open. First, the current system could be extended by incorporating federated learning to address the increasing concerns regarding data privacy and security in smart city IoT environments, as discussed in recent literature. Second, while the Selective Gradient Omission (SGO) mechanism effectively stabilized GAN training, exploring more advanced generative models like Diffusion Models could further enhance the quality of synthetic waste images. Additionally, the DQN-based sample selection strategy could be adapted into a multi-agent reinforcement learning (MARL) framework to coordinate multiple smart bins simultaneously in a real-time urban network. Finally, investigating the transition from a closed-set classification to an open-set recognition system would allow the model to identify novel or unknown waste materials that were not present in the initial training distribution, further improving the practical scalability of the system.

## Data Availability

The datasets generated or analysed during the current study are not publicly available, but are available from the corresponding author, Kimia Shirini on reasonable request.
